# Gene expression variability in mammalian embryonic stem cells using single cell RNA-seq data

**DOI:** 10.1016/j.compbiolchem.2016.02.004

**Published:** 2016-08

**Authors:** Anna Mantsoki, Guillaume Devailly, Anagha Joshi

**Affiliations:** The Roslin institute, University of Edinburgh, Easter bush campus, Midlothian EH25 9RG, UK

**Keywords:** Single cell RNA-seq, Embryonic stem cells, Transcription control, Gene expression variance, Coefficient of variance, Background

## Abstract

**Background:**

Gene expression heterogeneity contributes to development as well as disease progression. Due to technological limitations, most studies to date have focused on differences in mean expression across experimental conditions, rather than differences in gene expression variance. The advent of single cell RNA sequencing has now made it feasible to study gene expression heterogeneity and to characterise genes based on their coefficient of variation.

**Methods:**

We collected single cell gene expression profiles for 32 human and 39 mouse embryonic stem cells and studied correlation between diverse characteristics such as network connectivity and coefficient of variation (CV) across single cells. We further systematically characterised properties unique to High CV genes.

**Results:**

Highly expressed genes tended to have a low CV and were enriched for cell cycle genes. In contrast, High CV genes were co-expressed with other High CV genes, were enriched for bivalent (H3K4me3 and H3K27me3) marked promoters and showed enrichment for response to DNA damage and DNA repair.

**Conclusions:**

Taken together, this analysis demonstrates the divergent characteristics of genes based on their CV. High CV genes tend to form co-expression clusters and they explain bivalency at least in part.

## 1 Background

Transcription control is fundamental to mammalian system in defining gene expression programs that establish and maintain specific cell states during development. Any aberration to this process can result into disease phenotype. Microarray technology enables a genome-wide snapshot of the transcription landscape during development and disease by parallel quantification of large numbers of messenger RNA transcripts from different cell types and tissues (Schulze and Downward, 2001). This technology is widely used for differential gene expression analysis where studies are performed on a pool of hundreds of thousands of cells with an assumption that the variation across multiple samples from a cell population is largely due to experimental noise. Difference between mean values of gene expression is therefore the focus of such analyses and rarely the variability across the samples (Mar et al., 2011).

The breakthroughs in sequencing technology have now made it feasible to generate gene expression data for hundreds of individual cells from a cell population (Pan, 2014) providing new insights into early development (Tang et al., 2010) and differentiation (Shalek et al., 2013). Single cell RNA-seq sequencing is used for characterisation of hidden subpopulations of rare cell types, as closely related cells with the same phenotype can be discriminated to distinguish functionally each subgroup (Buettner et al., 2015). Importantly, the gene expression quantification by single-cell RNA-seq is consistent with the existing gold standards (Wu et al., 2013). The single cell gene expression data is variable between individual cells in contrast to the high concordance across replicates of populations of cells (Shalek et al., 2013). Though part of variation across individual cells is attributed to various confounding factors such as random technical noise mainly due to transcription bursts (Brennecke et al., 2013), protein fluctuations (Karwacki-Neisius et al., 2013) or mRNA fluctuations in response to cell cycle (Singh et al., 2013), there is no doubt about the biological relevance of variation in development (Xue et al., 2013), evolutionary adaptation, and disease (Feinberg and Irizarry, 2010).

Importantly, variation at a single cell level in genetically identical organisms in homogeneous environments indicates its role in generating diversity (Raj et al., 2010). Achieving such diversity is particularly important in the context of stem cells. The pluripotent state is a delicate equilibrium between the ability of self-renewal and differentiation, hence an imbalance (the variation of key pluripotency factors) could lead tipping the scale in favour of differentiation (Karwacki-Neisius et al., 2013). Accordingly, a high concordance was noted between global gene expression variability and heterogeneity of human pluripotency states (Mason et al., 2014). The differences between gene sets at the two ends of the spectrum of variation demonstrated that low variance genes were highly connected in the regulatory networks providing a causal hypothesis for their low variance (Mar et al., 2011). Highly variable genes, on the other hand, are thought to represent elements which fluctuate as the stem cell population moves between self-renewal and differentiation-potential (Mason et al., 2014). We collected single cell RNA sequencing data in human (Streets et al., 2014) and mouse (Yan et al., 2013) embryonic stem cells and identified ‘High CV’ (CV: Coefficient of Variation) gene sets. The multi-facetted bioinformatic analysis was based on CV enabled systematic characterisation of differences between the stable and variable gene sets.

## 2 Methods

### 2.1 Data collection and processing

Single cell RNA-seq data was obtained from Gene Expression Omnibus (GEO) database (Barrett et al., 2013) in fastq format. We downloaded 63 mouse single ES cell RNA-seq data (paired end) (GSE47835, SRP025171) (Streets et al., 2014) and 32 human single ES cell RNA-seq data (single end) (GSE36552, SRP011546) (Yan et al., 2013). After quality control using FastQC 0.11.2, alignment was done with TopHat 2.0.9 (Trapnell et al., 2009) using mm10 and hg38 as reference genomes and the GENCODE (Harrow et al., 2012) annotations (M4 and 22) for mouse and human respectively. Expression values for each single cell were calculated following the Cufflinks 2.2.1 (Trapnell et al., 2010) pipeline. The aligned reads were converted to expression values using the cuffquant command. Gene expression values for all single cell libraries were generated using the cuffnorm command with the default library normalization method (geometric). 39 mouse ES cells were selected for final analysis after discarding 24 cells due to low read quality or poor alignment scores.

### 2.2 Biological over technical variation threshold

From the initial normalized FPKM value matrix, we discarded the genes with 35 or more, zero expression values for mouse and 28 or more, zero expression values for human. We calculated the mean FPKM values (mean expression) across all cells for each of the remaining genes. We selected 229 (mESCs) and 217 (hESCs) highly expressed genes (>150 FPKM is each single cell) as highly confident sets. The remaining genes were sorted according to their mean expression levels and divided in windows of 1000 genes each (16 windows mouse, 19 windows human). The lowest windows (1259 genes in mouse, 1025 genes in human) were comprised of genes with the lowest mean expression levels, hence suffering from high levels of technical variation. We calculated the Pearson correlation coefficient for each pair of highly expressed genes with each gene in each window. For each window, (except the lowest one) we compared the distribution of correlation of all the gene pairs with the distribution of correlation of the lowest window using a *t*-test. We kept the genes with significantly higher correlation (probability distribution shifted to the right) compared to the lowest window (comparable to random noise). CV was determined as the ratio of standard deviation to mean for each gene across single cells.

### 2.3 Transcription factor enrichment

We used data from 49 and 99ChIP-seq experiments for transcription factors and chromatin remodellers in human and mouse embryonic stem cells respectively (Pooley et al., 2014). We selected peaks in promoter regions (+/− 1 kb from the TSS) of the two groups (High CV and Non High CV). For each promoter region, we also counted the total number of factors binding at the region.

### 2.4 miRNA target interactions

Data of miRNA target interactions in ES cells were retrieved from the ESCAPE database (Xu et al., 2013). From 693,552 interactions, we kept only the interactions that their target genes were in our one-to-one orthologs list and divided the number of miRNA interactions per gene in 3 bins (1–50, 51–100, >100).

### 2.5 Protein-protein interactions

Data of protein-protein interactions were retrieved from the ESCAPE database (Xu et al., 2013). One-to-one orthologs were used to map the genes for each category and for the total list of interactions. The number of proteins interacting with each gene were divided in four bins (1, 2, 3, >3).

### 2.6 Overlap with bivalent and active genes

We overlapped our genes with genes that were classified as bivalent or active (H3K4me3 marked) in human and mouse ES cells using published work from our lab (Mantsoki et al., 2015) and studied their differences at the level of CV.

### 2.7 Overlap with CpG islands and TATA box promoters

We calculated the overlap of the promoters of the genes with the CpG island regions as given from the UCSC tracks unmasked CpG islands for hg38 and mm10 (Karolchik et al., 2014). 2742 murine and 2010 human TATA-box motif promoters were retrieved from the Eukaryotic Promoter Database (Dreos et al., 2015).

### 2.8 Gene type classification

We calculated the fraction of genes that belonged to a specific gene type (from GENCODE annotation files). We selected only the types of genes with at least 30 genes in all the groups and plotted the CV for each category.

### 2.9 High variation threshold

For the sets of genes that were above the threshold of technical noise we calculated the coefficient of variation (CV) using the standard definition of ratio of the standard deviation to the mean, and divided them in four groups (quartiles) according to their CV. The High variation (High CV) genes were the ones that were falling in the fourth quartile of the CV. The rest of the genes were defined as Non High CV. Gene ontology enrichment was performed using DAVID (Dennis et al., 2003).

### 2.10 Correlation co-expression analysis

We calculated the Pearson correlation coefficient between all the pairs of High CV genes using FPKM values. We randomly permutated the FPKM values between cells for each gene to generate random data. The correlation distributions of High CV genes were significantly different (Wilcoxon test) than the random ones and we investigated their co-expression patterns by hierarchical clustering (flashClust package in R) visualised with heatmaps (heatmap.2 in R).

### 2.11 Conservation analysis

17,009 one-to-one orthologs from ensembl BioMart (Guberman et al., 2011) were used to calculate CV values in each species. After intersecting the orthologs with the 4000 genes (for both mouse and human) we end up with a gene set containing 2363 orthologous genes.

### 2.12 Topological associated domains

A lists of topological associated domains (TADs) for mouse and human ES cells (Dixon et al., 2012) was used to calculate the number of genes per TAD for the High CV and Non High CV genes in our analysis.

### 2.13 Bulk expression data

For the bulk RNA analysis we used 3 biological replicates of Microarray data from mouse ES cells (GSM1326660-2) (Zhang et al., 2014) and 4 biological replicates of RNA-seq data from hESCs (GSE33480) (Djebali et al., 2012).

### 2.14 Sequence conservation

The sequence conservation scores where obtained from PhyloP100way (Human) and PhyloP60way (Mouse) tracks available at UCSC.

## 3 Results

### 3.1 Correlation based approach to identify genes with significant biological variation in mammalian single embryonic stem cell RNA-seq data

**Fig. 1:**
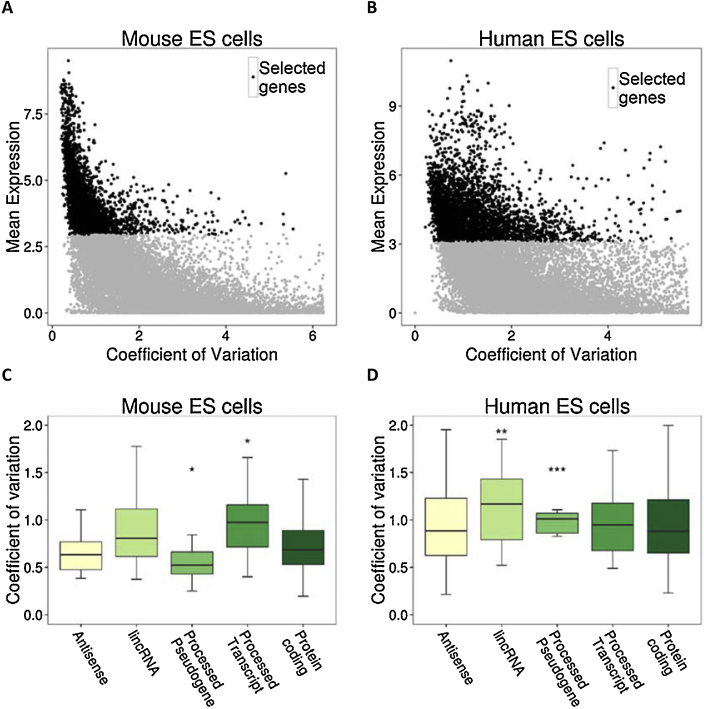
Correlation based approach for the identification of genes above the threshold of technical variation (A, B) Scatterplots showing genes according to their mean expression (log (mean FPKM + 1)) and coefficient of variation in mouse and human ES cells. The genes highlighted in black were chosen for the analysis, since they were more correlated with the highly expressed genes. (C, D) Gene types in mouse and human ES cells and their respective CV levels (shown only the genes types that were found in 30 genes or more).

To study the gene expression variability across individual cells, we collected RNA sequencing data for 32 human and 39 mouse single ES cells. After normalising the data across cells, we calculated FPKM values for 43,345 mouse and 60,468 human GENCODE (Harrow et al., 2012) genes in each single cell. Single cell sequencing data suffers from low genome coverage and high amplification bias. These biases contribute to technical variation (noise) which hinders capturing biological variation across individual single cells. To distinguish the genes with significantly higher biological variation over technical variation, we developed a correlation-based approach. As highly expressed genes tend to have lower technical noise, we selected top 229 (mouse) and 217 (human) highly expressed genes (see Section 2) across single cells. We then binned the genes based on their mean expression level. We calculated the correlation of genes in each bin with the highly expressed genes. We noted that technical noise was inversely related to the mean expression of gene sets i.e. higher the gene expression, lower the technical noise. We selected a threshold on expression value where the correlation with highly expressed genes was statistically significant over correlation with gene sets with technical noise (see Section 2). This procedure resulted in selection of 4229 genes over 2.9 mean expression threshold (log(FPKM + 1)) in murine ES cells (Figs. 1 A and S1 ) and 4217 genes over log mean expression threshold of 3.1 in human ES cells (Figs. 1 B and S2) with significantly higher biological noise than technical noise.

Gene expression variability was negatively correlated with the mean expression level i.e. highly expressed genes had low CV while lowly expressed genes spanned a wide spectrum on CV range (Fig. 1A and B). The functional enrichment of low CV genes resulted in enrichment for cell cycle functional category specifically the ‘M phase’ of mitotic cell cycle for both human and mouse ES cells. We further calculated the functional enrichment for highly expressed genes irrespective of CV values. They were also enriched for cell cycle functional category in both human and mouse ES cells. We therefore inferred that highly expressed genes tend to have low CV and are involved in cellular functions such as cell cycle.

We further checked if different gene categories provided by GENCODE (Harrow et al., 2012) demonstrate variability comparable to protein coding genes (Fig. 1C and D). The lincRNAs had higher CV values in both human (*t*-test, *P*-value < 0.01) and mouse ES cells (*t*-test, *P*-value < 0.05). An overwhelming fraction of murine processed pseudogenes had low CV (*t*-test, *P*-value < 0.05). In contrast, a significant fraction of human processed pseudogenes had CV higher than protein-coding genes (*t*-test, *P*-value < 0.001). Processed transcripts and antisense transcripts on the other hand show no significant difference, possibly due to low sample numbers.

### 3.2 Genes occupied by many transcription factors have a lower CV

**Fig. 2:**
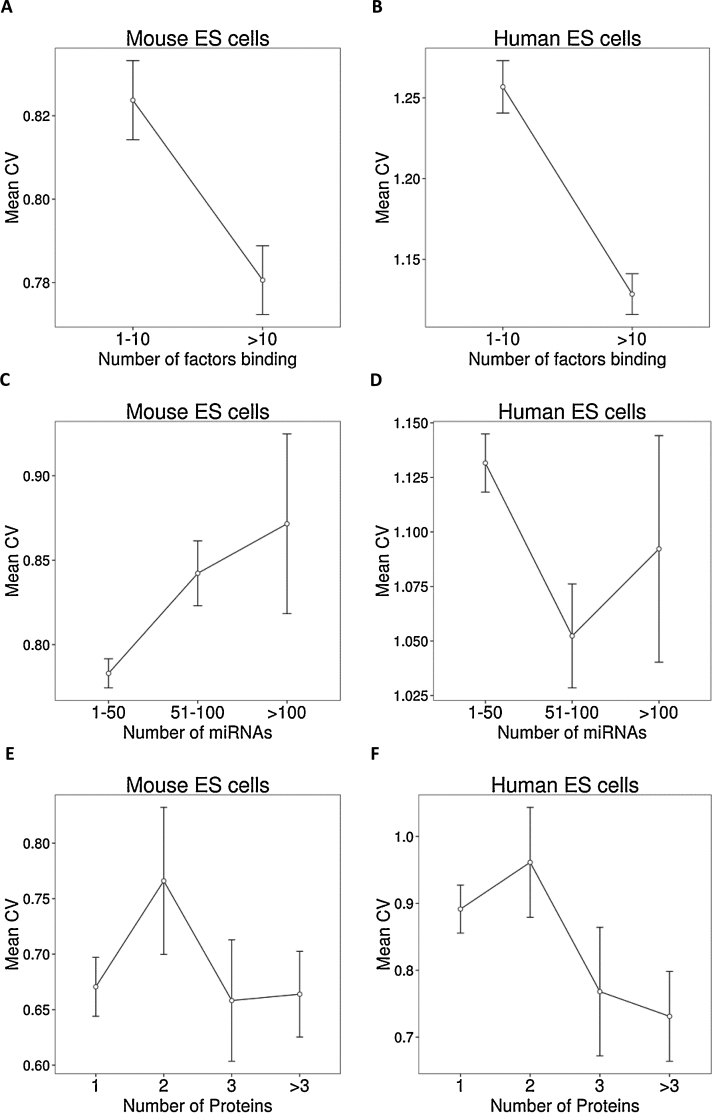
Mean CV levels according to quantification of transcription factors, miRNA targets and protein-protein interactions. (A, B) Transcription and epigenetic factor occupancy (number of factors binding) at the promoters of genes is inversely correlated with their mean CV in mouse (99ChIP-seq TFs) and Human (49ChIP-seq TFs) ES cells. (C, D) Bins of miRNAs targeting each gene and their responding mean CV levels (only interactions with genes in orthologs one2one list have been used) in mouse and human ES cells. (E, F) Genes (only interactions with genes in orthologs one2one list have been used) with known protein–protein interactions for mouse and human ES cells and their responding mean CV levels.

In order to study the level of transcription control among the promoters, we calculated the number of factors binding at each promoter using ChIP sequencing compendia for transcription and epigenetic factors in human and ES cells (Pooley et al., 2014). The mean CV for genes bound by less than 10 factors was significantly higher than the mean CV for genes bound by more than 10 factors in both human (*t*-test, *P*-value < 0.001) and mouse (*t*-test, *P*-value < 0.001) ES cells (Fig. 2A and B). This result was consistent when average binding of individual factors was tested as well i.e. genes more likely to be bound by more factors tended to have low CV. We obtained the number of putative binding sites of transcription factors in gene promoters from UCSC. Again, number of putative binding sites varied inversely with the CV value (Fig. S3).

To test the regulation at post-transcriptional level, we collected putative miRNA targets predicted by four miRNA prediction methods (Xu et al., 2013). Unlike TF targets, there was no bias towards the number of miRNA targets with respect to their mean CV, either in human or mouse ES cells (Fig. 2C and D).

Finally we collected known protein–protein interactions (PPI) in mouse and human ES cells (Xu et al., 2013) and calculated the number of known interacting partners for each of the genes. Similarly to miRNA targets, there was no statistically significant difference between the mean CV values based on the number of interacting partners at protein level in either human or mouse ES cells (Fig. 2E and F).

### 3.3 High expression variability genes correlate with DNA repair and bivalency

The activity of signalling pathways such as TGF-β-related signalling pathways are thought to prime cells for differentiation contributing to the heterogeneity between cells in ES cells (Galvin-Burgess et al., 2013). The CV value did not distinguish any particular signalling pathway. The differences in micro-environments sensed by the signalling pathway can manifest in large expression changes of its downstream target genes. We therefore tested whether transcription factor and chromatin remodeller binding prefers or avoids gene promoters based on their CV measure using the ChIP sequencing data compendium for 49 and 99 factors in mouse and human ES cells respectively (Pooley et al., 2014). Unsurprisingly, many promoter specific factors such as E2F1, TAF1, and YY1 did not show any bias for the CV. High CV genes in mouse ES cells showed an exclusive binding preference of the following four factors: NCOA3 (Hypergeometric test, *P*-value < 0.0001), p300 (Hypergeometric test, *P*-value < 0.0001), MCAF1 (Hypergeometric test, *P*-value < 0.01) and p53(Hypergeometric test, *P*-value < 0.05).

NCOA3 is a nuclear receptor activator with a histone acetyltransferase activity, recruiting the chromatin modifying proteins p300, CARM1 and CBP at the *Nanog* locus (Wu et al., 2012). NCOA3 is thought to be critical for both the induction and maintenance of pluripotency, acting as an essential Esrrb coactivator (Percharde et al., 2012). ESRRB is downstream of NANOG which is a direct target of TGF-β mediated SMAD signalling (Xu et al., 2008). NANOG targets did not show any bias with respect to CV.

MCAF1 is a nuclear protein associated with heterochromatin, shown to colocalize with SETDB1 in PML bodies (Sasai et al., 2013). PML is a protein involved in the senescence pathway through the p53 signalling, and its overexpression leads to premature senescence (Pearson et al., 2000). p53 is a sequence specific transcription factor with tumour suppressor activity, regulating cell cycle arrest, apoptosis, senescence and stem cell differentiation, acting as an activator or suppressor of its downstream targets (Vousden and Prives, 2009). Upon DNA damage, p53 activates differentiation associated genes and represses self-renewal genes, affecting the status of ES cells (Li et al., 2012).

Accordingly, high CV genes showed enrichment for biological processes such as cellular response to stress (adjusted *P*-value < 10^−4^), response to DNA damage stimulus (adjusted *P*-value < 10^−3^) and DNA repair (adjusted *P*-value < 10^−3^) in both murine and human ES cells.

**Fig. 3:**
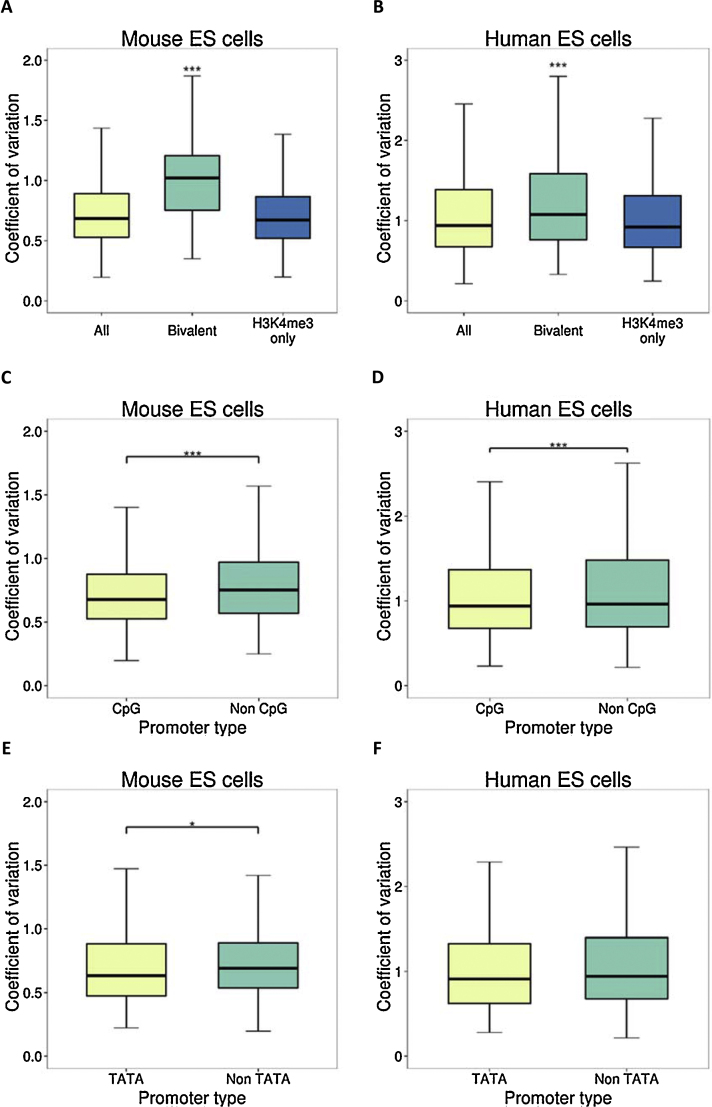
Chromatin modifications and sequence features of genes and their corresponding coefficient of variation. (A, B) Overlapping genes with bivalent and active (H3K4me3 marked) gene promoters in response to their CV, in mouse and human ES cells. Bivalent genes show significantly higher CV levels than all the promoters (irrespective of overlap) and the active promoters (pairwise *t*-test, *P*-value < 0.001) (C) CV levels of genes having a CpG island and a non- CpG island promoter. (D) CV levels of genes having a TATA box and a non-TATA box promoter.

The genes overlapping with bivalent promoters had statistically significant higher CV values than the ones overlapping with the active promoters (presence of H3K4me3 and absence of H3K27me3 modifications) in both human (Hypergeometric test, *P*-value < 0.001) and mouse (Hypergeometric test, *P*-value < 0.001) ES cells (Fig. 3A and B). Genes with high CV showed a weak functional enrichment for embryonic development and transcription control; the functional categories associated with bivalent genes (Bernstein et al., 2006).

As specific promoter structures such as presence of TATA boxes have been previously associated with genes with highly fluctuating single-cell levels within populations (Choi and Kim, 2009), we calculated TATA and CpG island fraction for all human and mouse promoters (−/+ 1Kb from TSS). The CpG-rich promoters showed lower CV values than the CpG-poor promoters and the difference was statistically significant in both human and mouse ES cells (*t*-test *P*-value < 0.001) (Fig. 3C and D). Unlike CpG promoters, TATA box promoters could not be distinguished based on the CV value (Fig. 3E and F).

### 3.4 High CV genes form dense highly co-expressed clusters

**Fig. 4:**
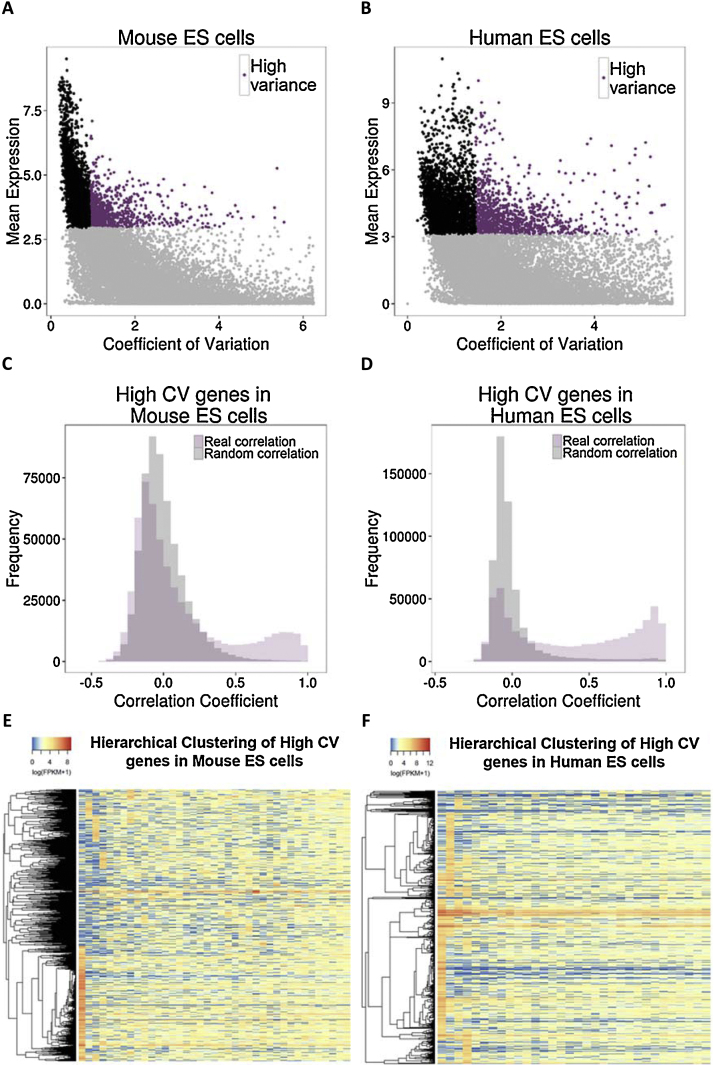
High variance genes are more correlated than expected by chance (A, B) Scatterplot of genes in response to their CV and mean expression. Highlighted in purple are the High variance genes, selected based on their CV (CV value greater than the third quartile of the distribution). (C, D) Correlation coefficient distributions for the High variance (High CV) genes in mouse and human ES cells (statistically significant difference (*p* < 0.001, Wilcoxon test) between the real and random distributions). (E, F) Heatmaps of gene expression (in log(FPKM + 1) values) for the High variance genes (High CV) in mouse and human ES cells. (For interpretation of the references to colour in this figure legend, the reader is referred to the web version of this article.)

In order to study the characteristics of genes with high variability, we defined genes with CV value greater than 0.92 (3rd quartile value) as High CV in mouse (Fig. 4A) and genes with CV value greater than 1.45 (3rd quartile value) in human ES cells (Fig. 4B). We then checked whether the expression of High CV genes varies concordantly across single cells by calculating Pearson’s correlation coefficient between all pairs of High CV genes. A subset of High CV genes were significantly more correlated with each other compared to expected from a random permutation (Fig. 4C (mouse) and D (human)).

The highly correlated network (Pearson’s correlation coefficient >0.95) of High CV genes grouped them mainly into only few tightly co-expressed clusters in both human and mouse ES cells (Figs. S4 and S5). Interestingly, the genes in each cluster were highly expressed only in one individual cell (Fig. 4E (mouse) and F (human)). We firstly confirmed that these single cells (e.g. single cell 24 and 26 in humans) did not suffer from poor technical quality of samples (Fig. S6). We also removed these two cells and redefined the High CV gene set (Fig. S7) to find a similar result. This assured that the significant co-expression among High CV genes is not an artefact of few aberrant single cells.

The co-expressed genes derived from large-scale analyses of mammalian expression data have demonstrated that neighbouring genes tend to have similar expression profiles (Lercher et al., 2002). As high CV genes formed tight co-expression clusters, we checked whether they tend to be in gene neighbourhoods with each other compared to other genes. We did not observe any tendency of genes clustering based on CV value. We also checked whether there was any bias towards similar CV genes co-existing in topological associated domains (TADS) inferred from Hi-C chromatin capture data in human and mouse ES cells (Dixon et al., 2012). There was no bias towards associating similar CV value genes with same TADS. Also, tightly co-expressed High CV genes in each cluster were not specifically enriched for any biological process nor primed for specific lineage.

### 3.5 CV values are conserved across species

**Fig. 5:**
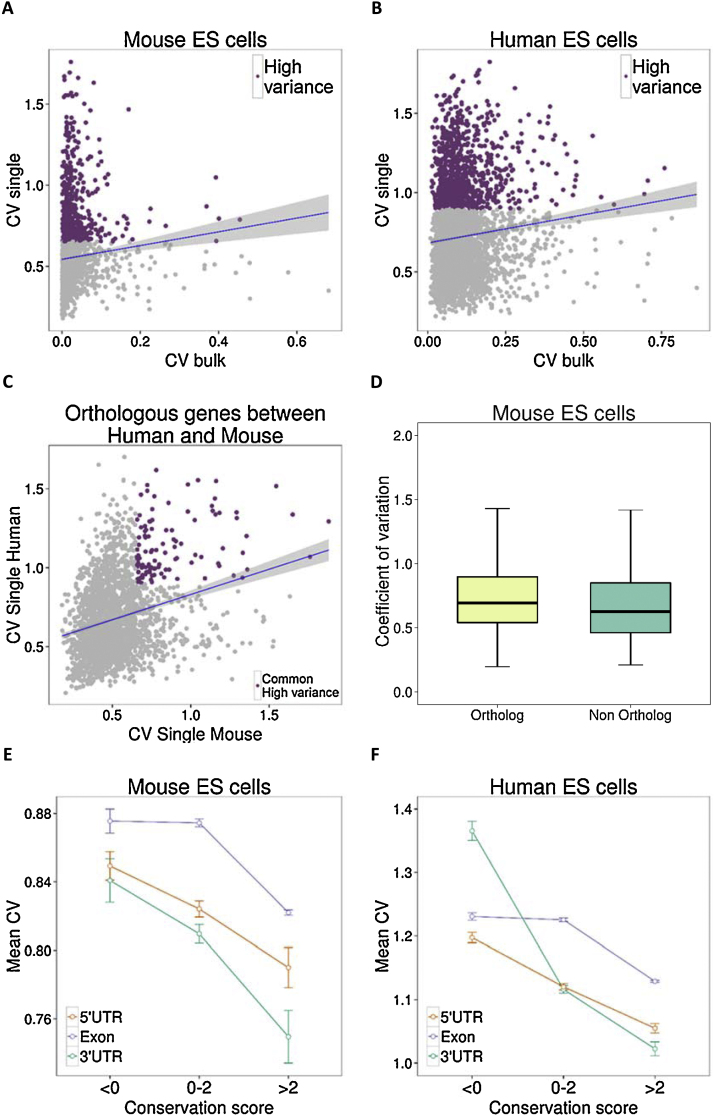
Conservation of expression variability across technologies and species. (A, B) Scatterplot of CV values in a bulk expression study against CV values in a single cell RNA–seq study in mouse and human ES cells. There is a positive correlation between the CV values of the two technologies (Pearson’s *r* = 0.06 for mouse, *r* = 0.09 for human). (C) Scatterplot of CV values of orthologous genes between human and mouse from single RNA-seq studies in ESCs. There is a positive correlation of CV values between species (Pearson’s *r* = 0.23) and 10% of High CV genes (highlighted in purple) are conserved as highly variant between species (D) Boxplot of CV values of orthologous and non-orthologous genes between human and mouse in ESCs (3675 orthologs and 554 non-orthologs out of 4229 genes in our analysis). (E, F) Sequence conservation scores and their corresponding mean CV values for 5′UTR, Exons and 3′UTRs in mouse and human ES cells. (For interpretation of the references to colour in this figure legend, the reader is referred to the web version of this article.)

In order to check whether the CV values are conserved between bulk and single cell experiments, we obtained gene expression values for bulk RNA in human and mouse ES cells. The CV values of genes from single cells and bulk RNA showed no correlation in both human (Pearson’s correlation coefficient *r* *=* 0.09) and mouse (Pearson’s correlation coefficient *r* *=* 0.06) ES cells (Fig. 5A and B).

To test whether gene expression variability from single and bulk RNA-seq is conserved across species, we collected one-to-one orthologs between human and mouse (Guberman et al., 2011). The gene expression tends to be conserved across species for single (Pearson’s correlation coefficient *r* *=* 0.23) (Fig. 5C) i.e. orthologs of genes with lower CV in mouse are more likely to have lower expression variance across human single ES cells and vice versa. We confirmed that the distribution of CV values for orthologous genes in mouse was not significantly different from mouse-specific genes (Fig. 5D). We further checked whether the expression conservation goes hand-in-hand with the conservation at the sequence level. Indeed, sequence conservation showed a negative correlation with the CV values in both human and mouse ES cells in their 5′UTR, their 3′UTR and their exons (Fig. 5E and F). Thus tight regulation of gene expression level is a feature that appears to be conserved and selected during evolution.

## 4 Conclusion and discussion

Single cell RNA-seq data holds a great promise for studying variability across individual cells with the hindrance of large technical noise inherent to these data. Though availability of data from a limited number of cells (32 in human, 39 in mouse) could influence the results, it has been recently shown that 30 cells is the lower limit of sample size to sufficiently converge to the complexity of large cell populations (Marinov et al., 2014). We used a correlation based approach to define a set of genes with biological variation significantly higher than technical variation across single cells. We then studied the characteristics of expression variability for 4217 genes in human and 4229 genes in mouse single ES cells, where the estimated biological variability was significantly greater than the technical variability. We noted that highly expressed genes tended to have lower CV (Fig. 1A and B). Since ES cells are not synchronized in their cell cycle and can belong to different development stages, we specifically looked whether genes with high CV were developmental stage specific or involved in specific function, but did not find a strong evidence for it.

High CV genes form co-expression clusters. Tightly co-expressed High CV genes in each cluster were highly expressed only in one or a few single cell(s) and genes in each cluster were not specifically enriched for any biological process. This fits with the notion of pluripotent cells to alternate between different transient and reversible cell states without showing any functional bias or lineage priming. High CV genes showed enrichment for response to DNA damage and DNA repair and were exclusively bound by regulators of DNA damage and senescence pathways like MCAF1 and p53. They also showed significant overlap with bivalent genes in human and mouse ES cells. This confirms that at least a subset of bivalent genes can indeed be attributed to heterogeneity in ES cells.

Though many characteristics of CV genes are conserved across species, there are some differences. Interestingly the vast majority of murine processed pseudogenes have lower CV than protein-coding genes while human processed pseudogenes have higher CV than protein-coding genes. Processed pseudogenes have recently been demonstrated to play a regulatory role by competing with other genes for the binding of small RNAs (Poliseno et al., 2010). This potential species specific regulatory aspect needs to be explored in detail.

Taken together, genes with lower CV tend to be highly expressed, tightly regulated at transcriptional level as they are likely to be central to many cellular processes. High CV genes, on the other hand, are highly expressed only in individual single cells which possibly partly explains the bivalent genes (with both active and inactive chromatin status) observed in bulk studies.

## Conflict of interests

The authors declare no completing interests.

## Authors’ contributions

A.M. collected the data, performed the analysis and helped write the manuscript, G.D. helped perform the analysis, and A.J. conceived the idea, supervised the project and wrote the manuscript.
