# Supplementary material for: Gene expression variability in mammalian embryonic stem cells using single cell RNA-seq data
Source: Comput Biol Chem. 2016 Aug;63:52–61. doi: 10.1016/j.compbiolchem.2016.02.004 (PMC5012374; doi:10.1016/j.compbiolchem.2016.02.004)
Supplement: Supplementary file 1 [file mmc1.docx]

**Supplementary material for**

**Gene expression variability in mammalian embryonic stem cells using single cell RNA-seq data**

Anna Mantsoki^1^, Guillaume Devailly^1^, Anagha Joshi^1§^

^1^The Roslin institute, University of Edinburgh, Easter bush campus, Midlothian, EH25 9RG.

^§^Corresponding author

| **Mouse ES Cells** | |
| --- | --- |
| 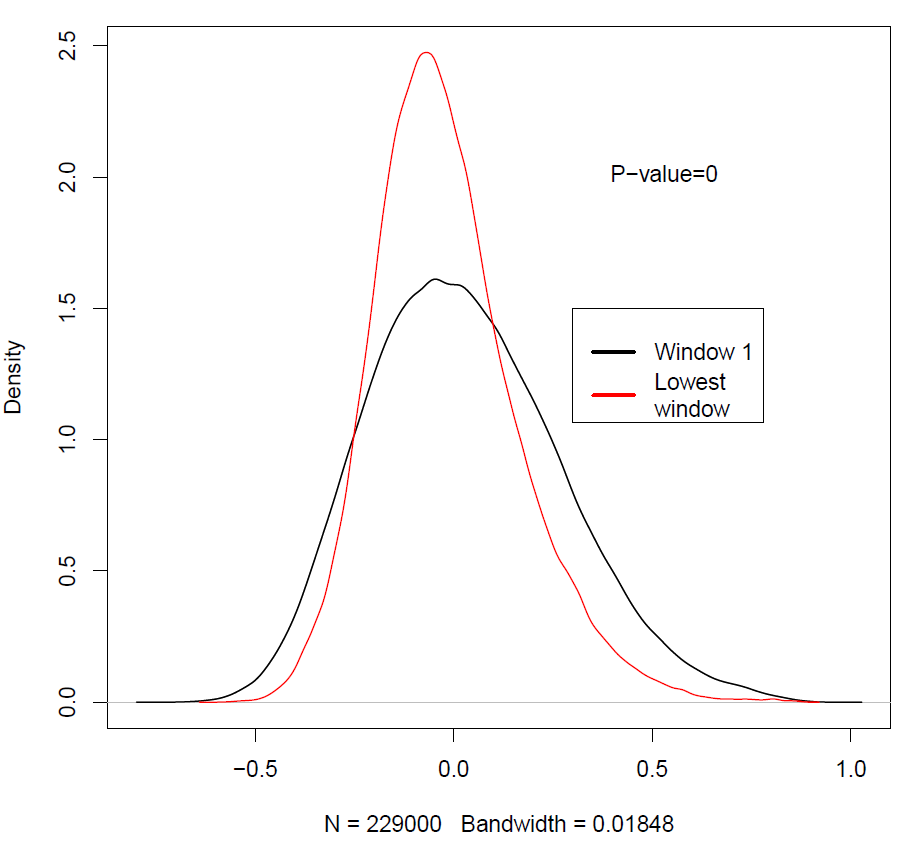 | 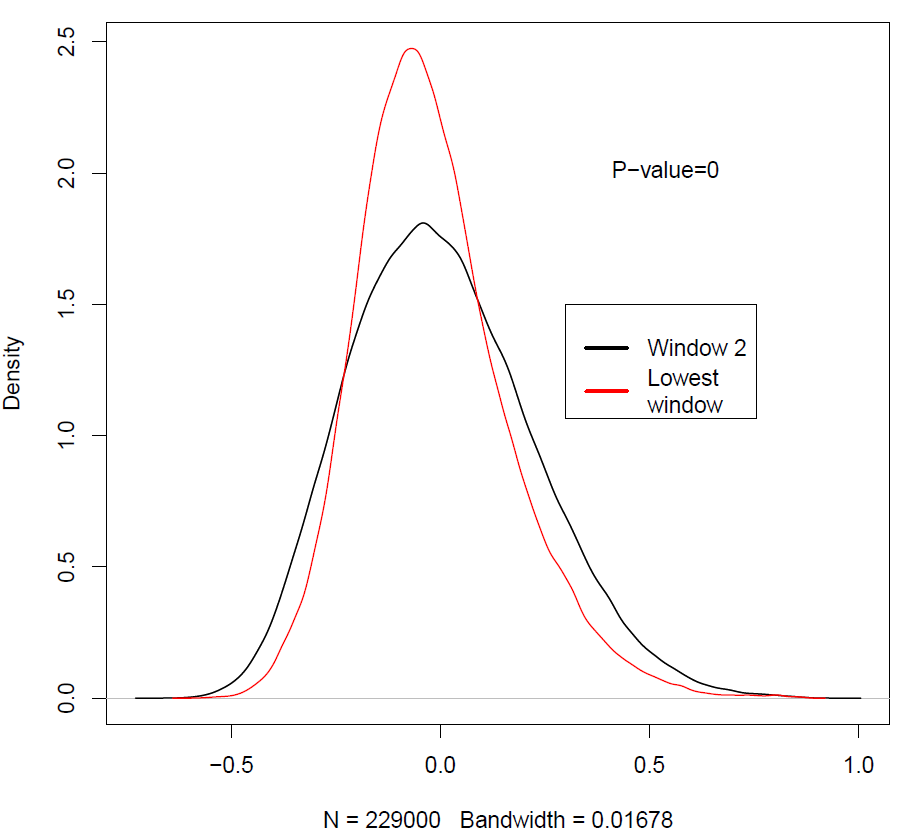 |
| 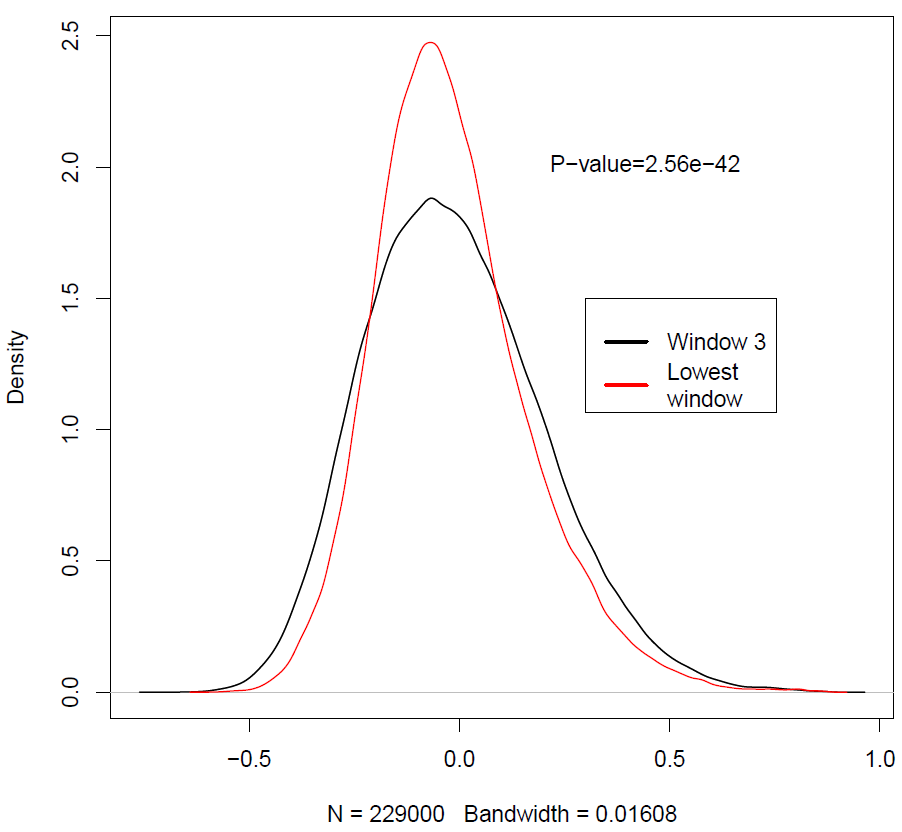 | 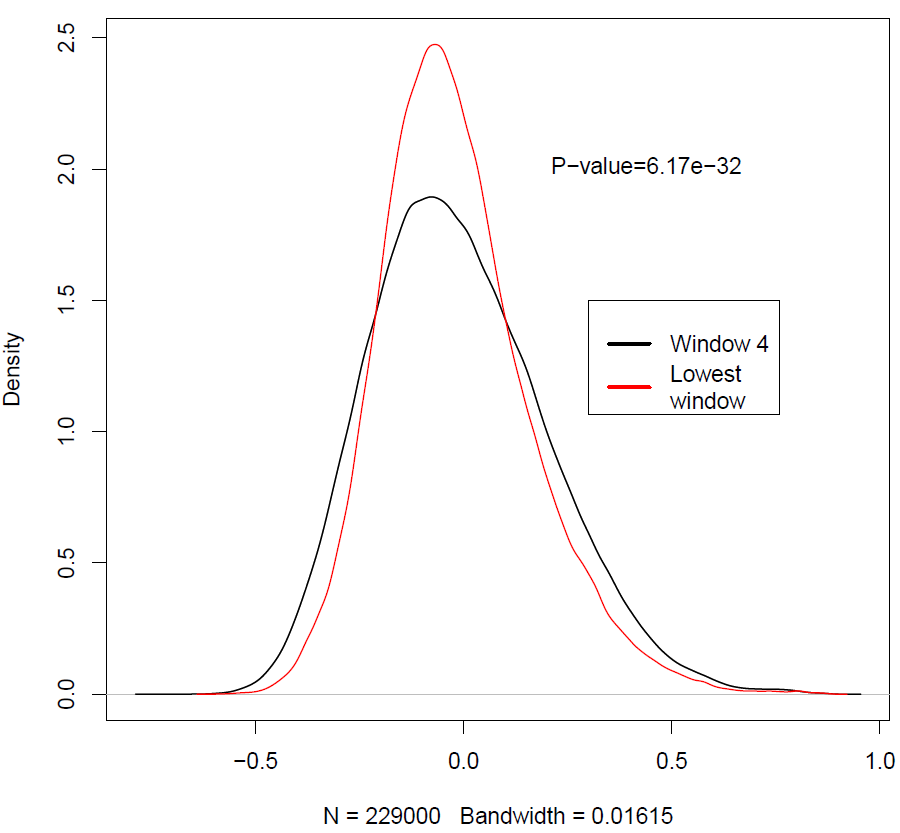 |
| 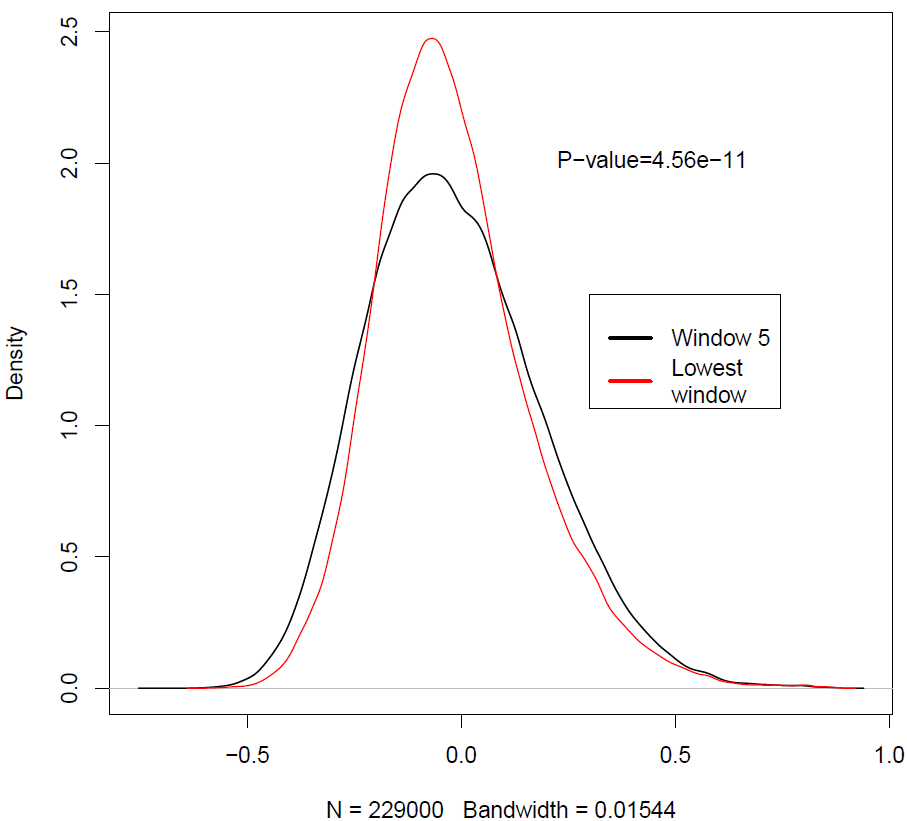 | 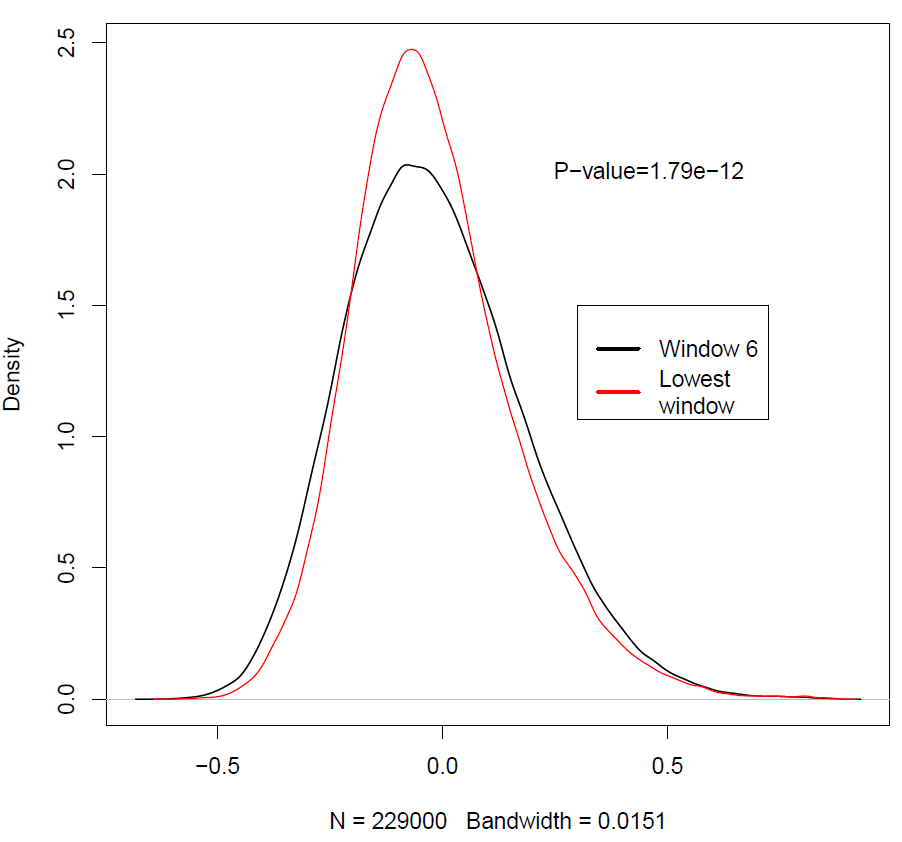 |
| 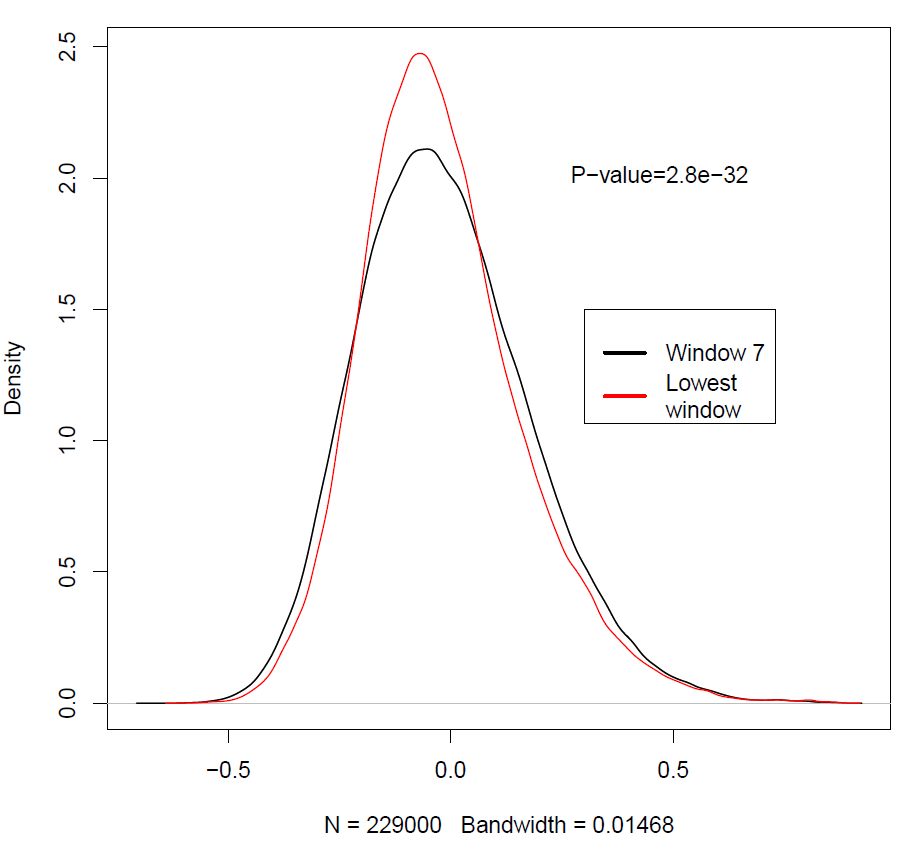 | 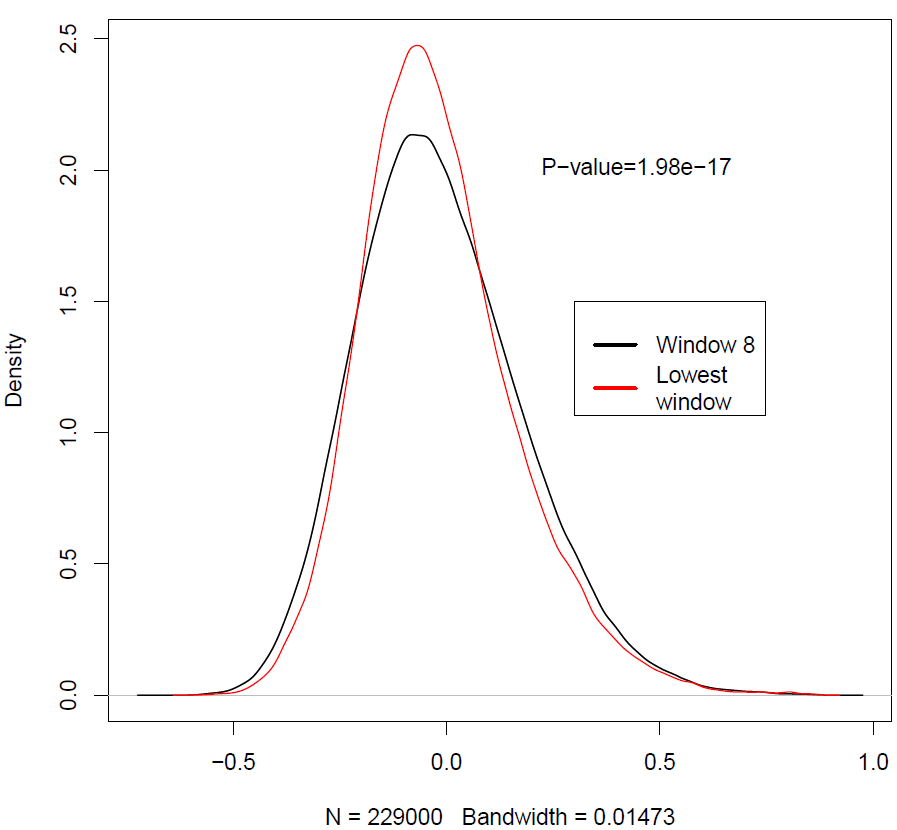 |
| 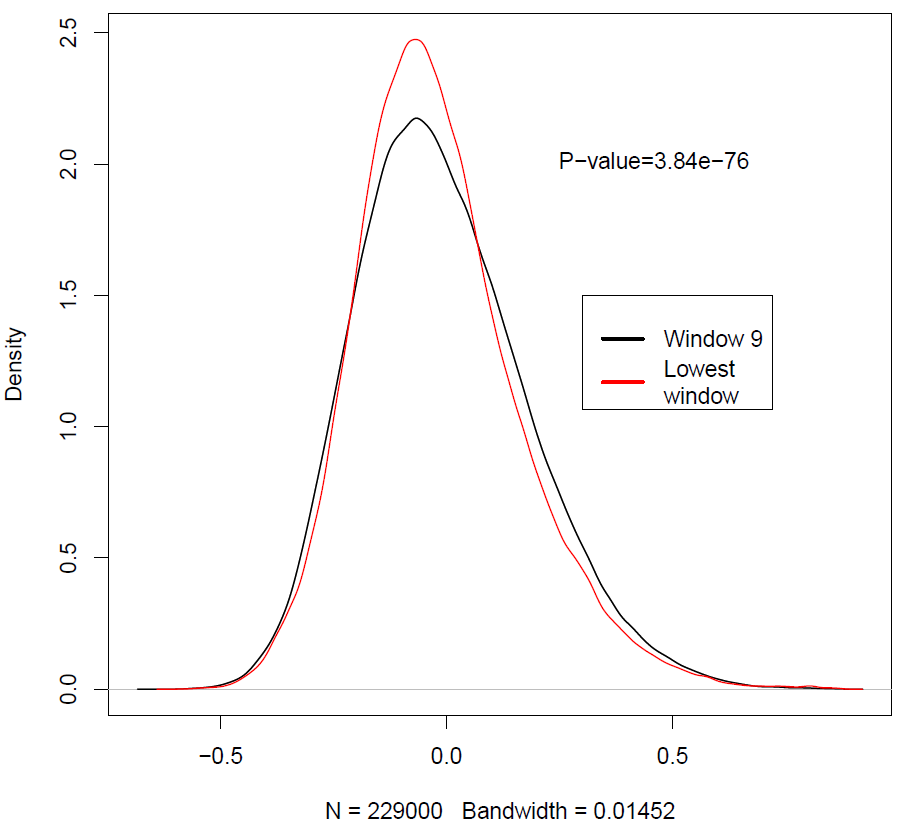 | 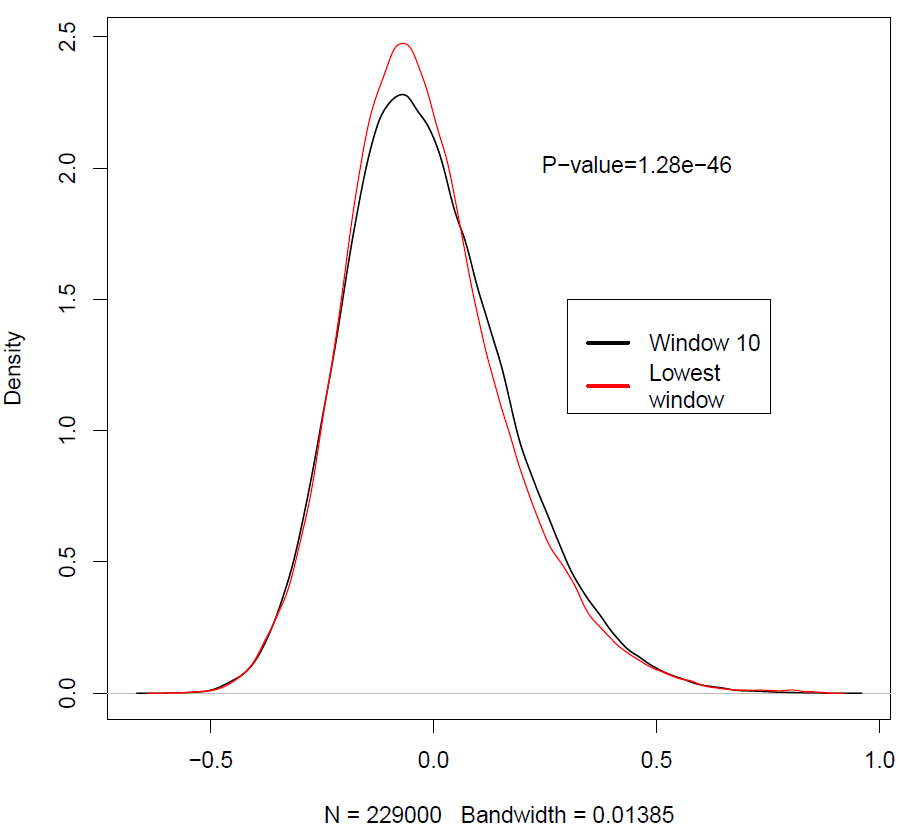 |
| 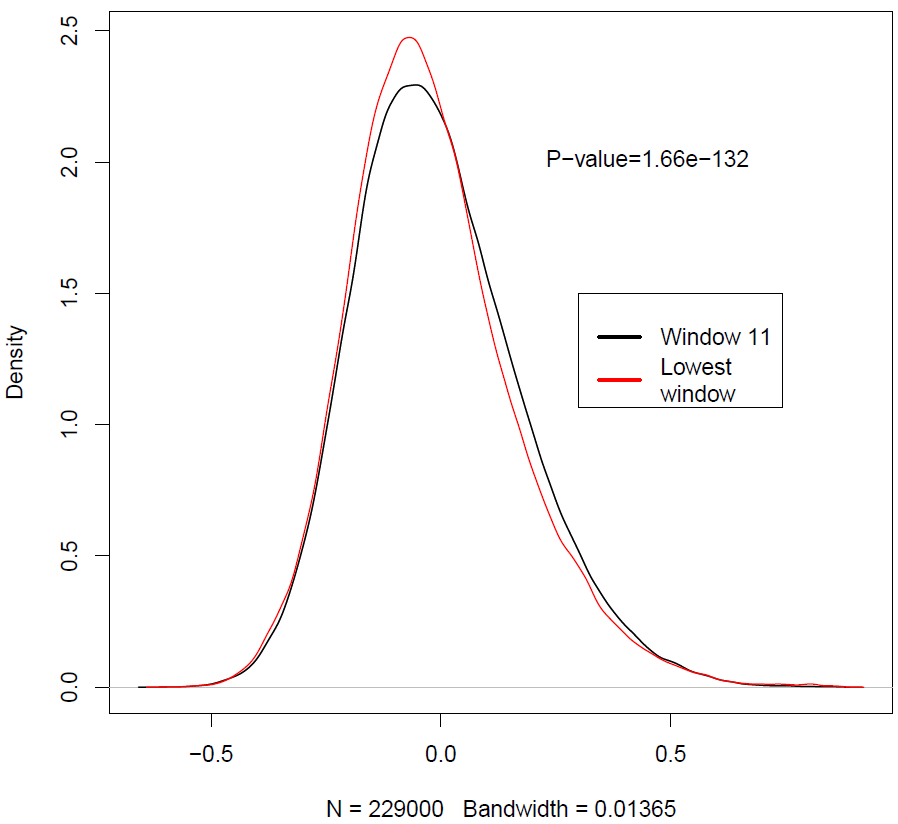 | 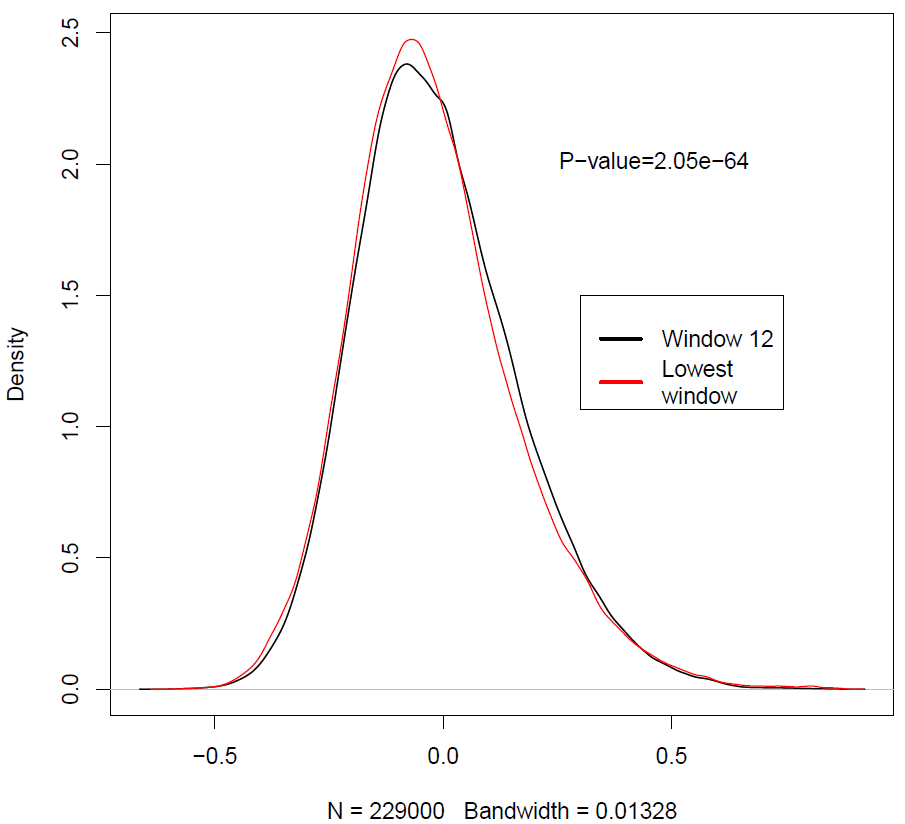 |
| 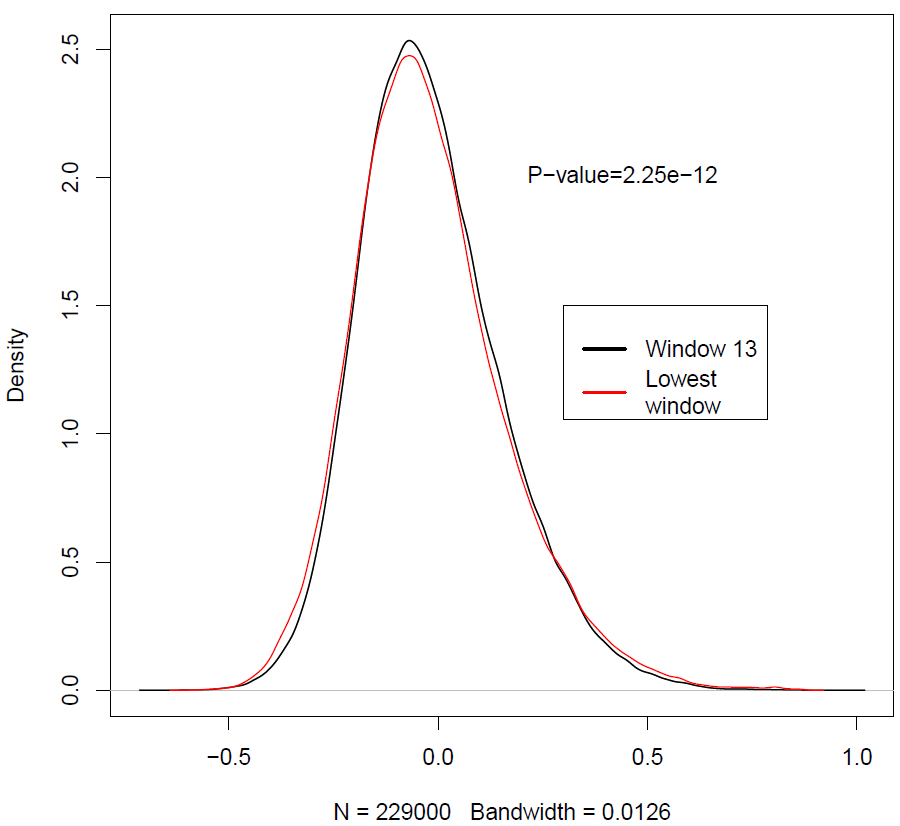 | 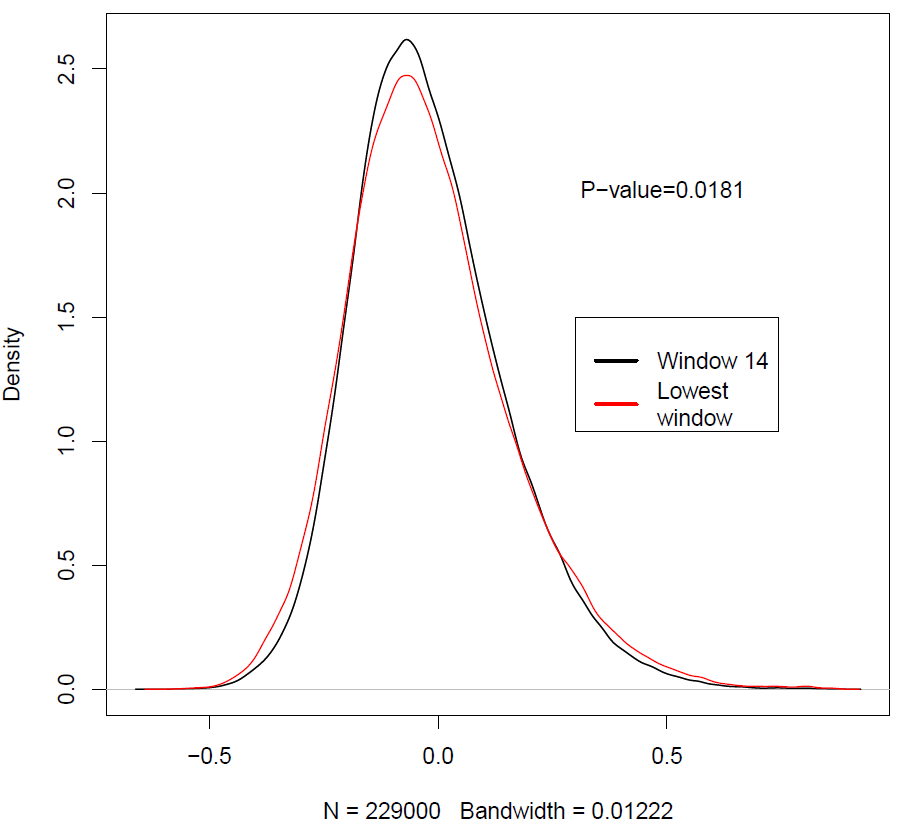 |
| 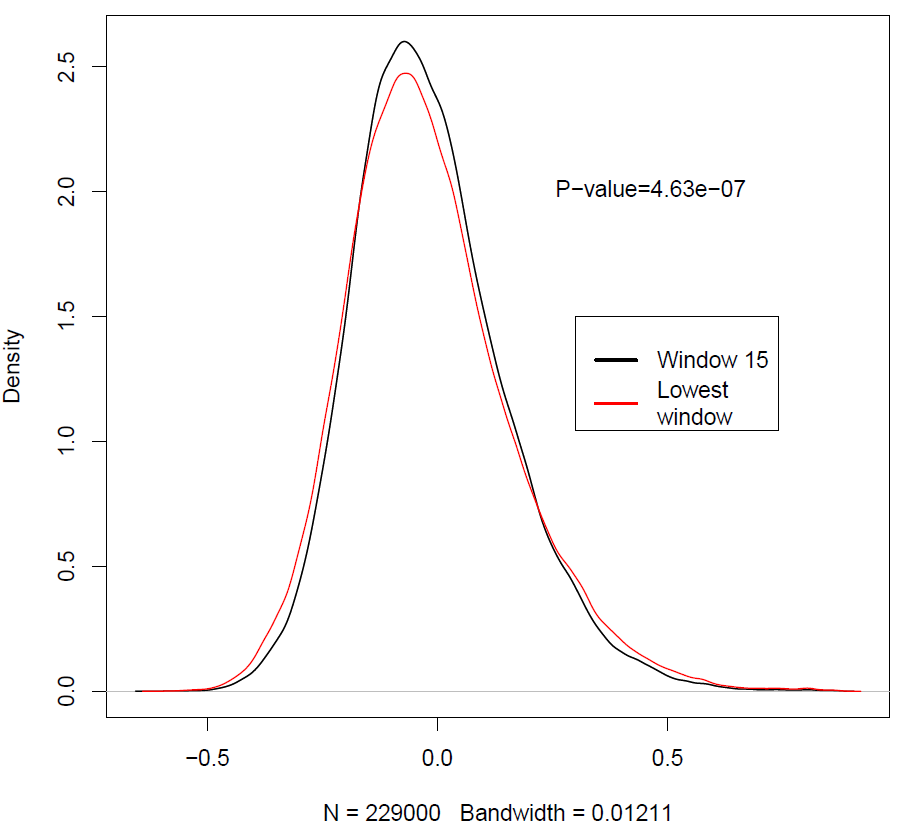 |  |

**Figure S1.** Distributions of correlation of high expression genes with genes in 15 windows of mean expression compared with the lowest mean expression window, in Mouse ES cells. The genes of Windows 1 to 4 were chosen as the ones that are above the threshold of technical variation.

| **Human ES Cells** | |
| --- | --- |
| 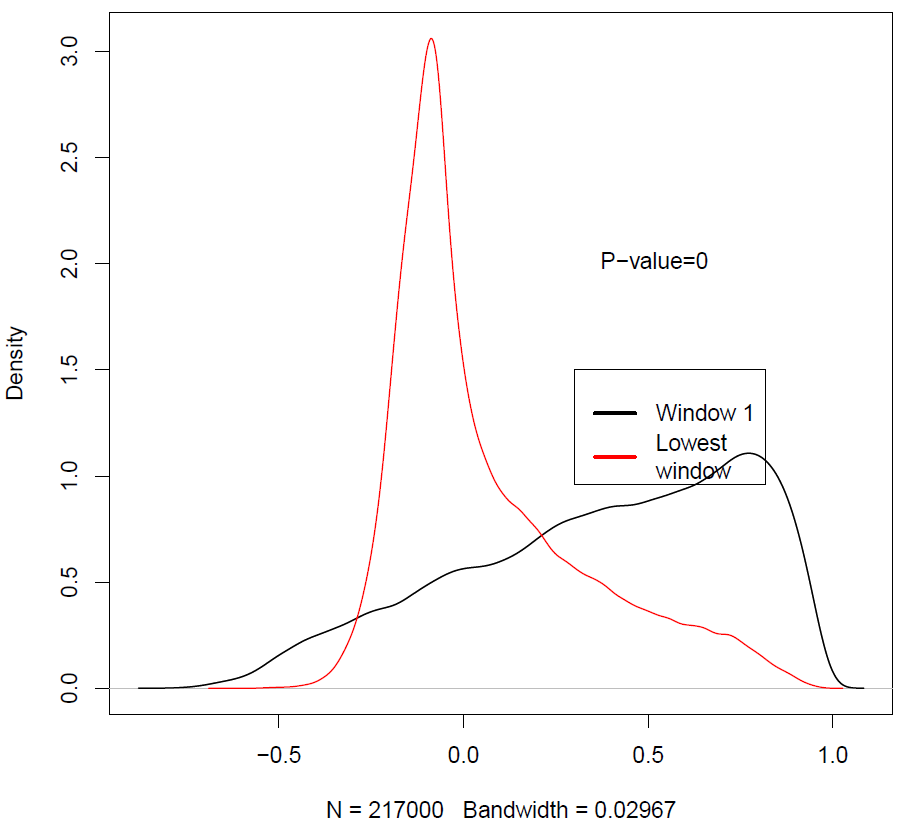 | 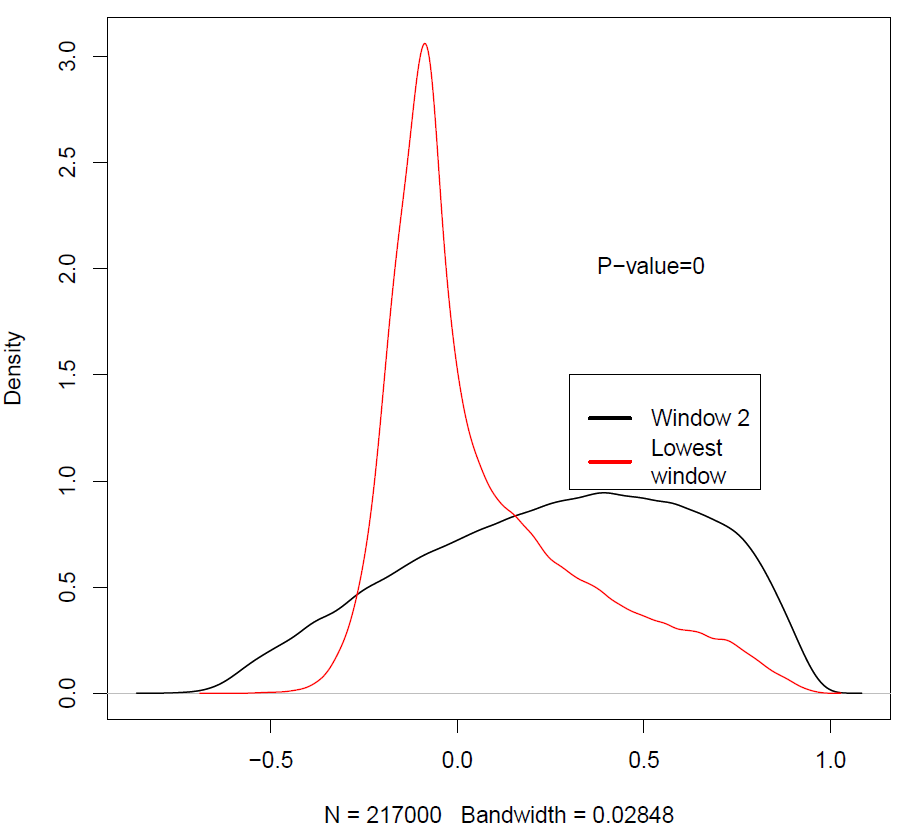 |
| 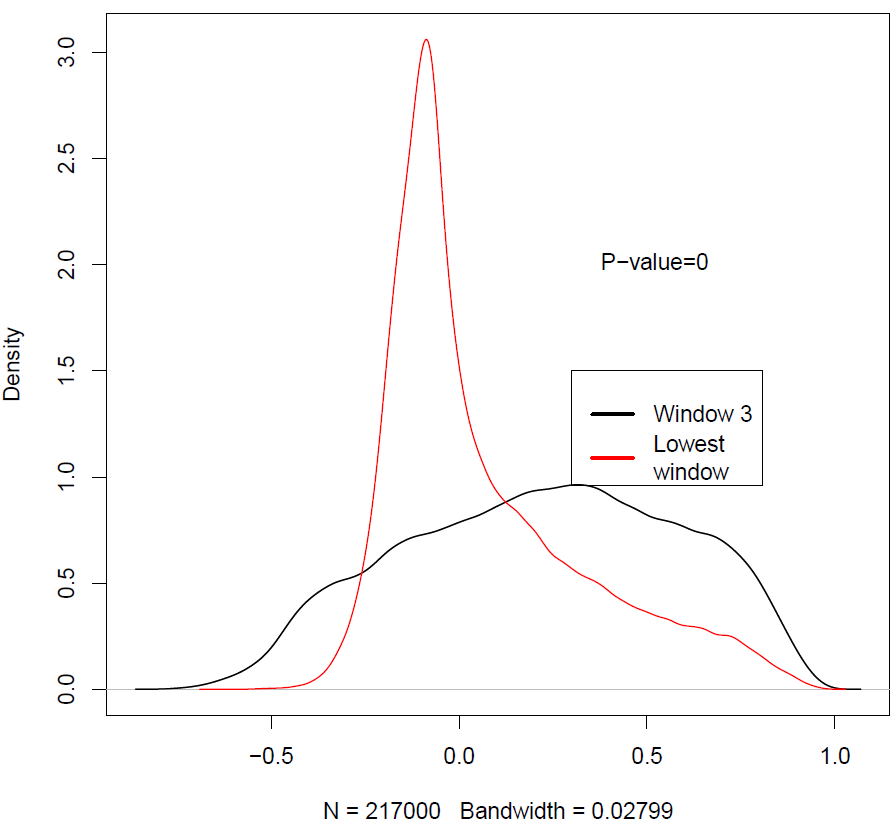 | 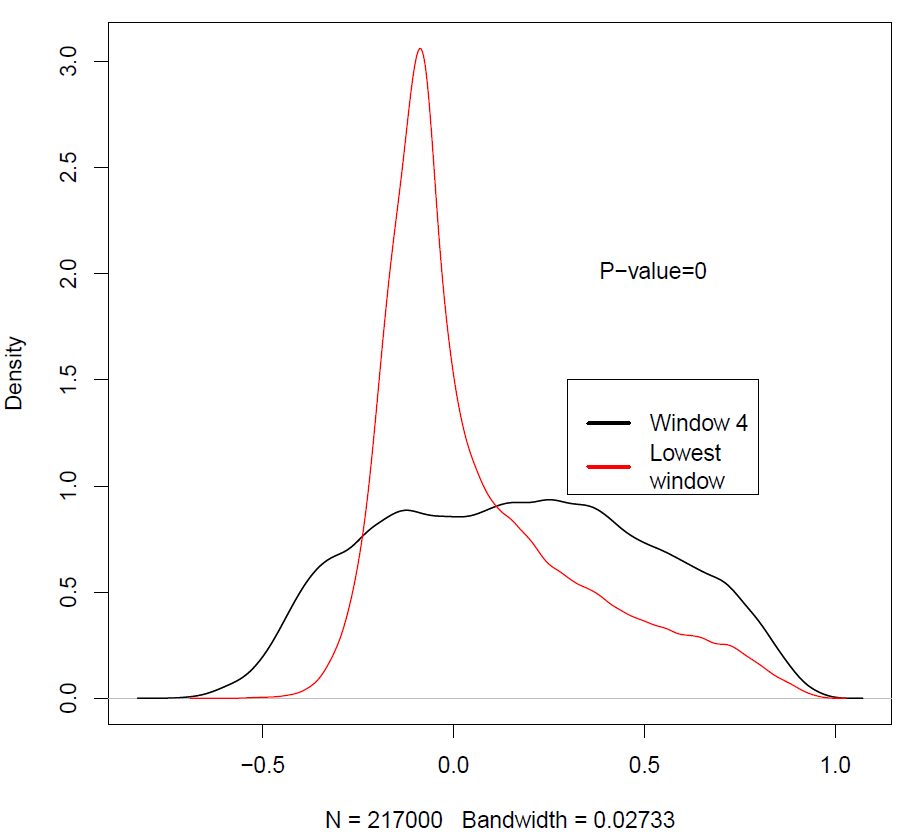 |
| 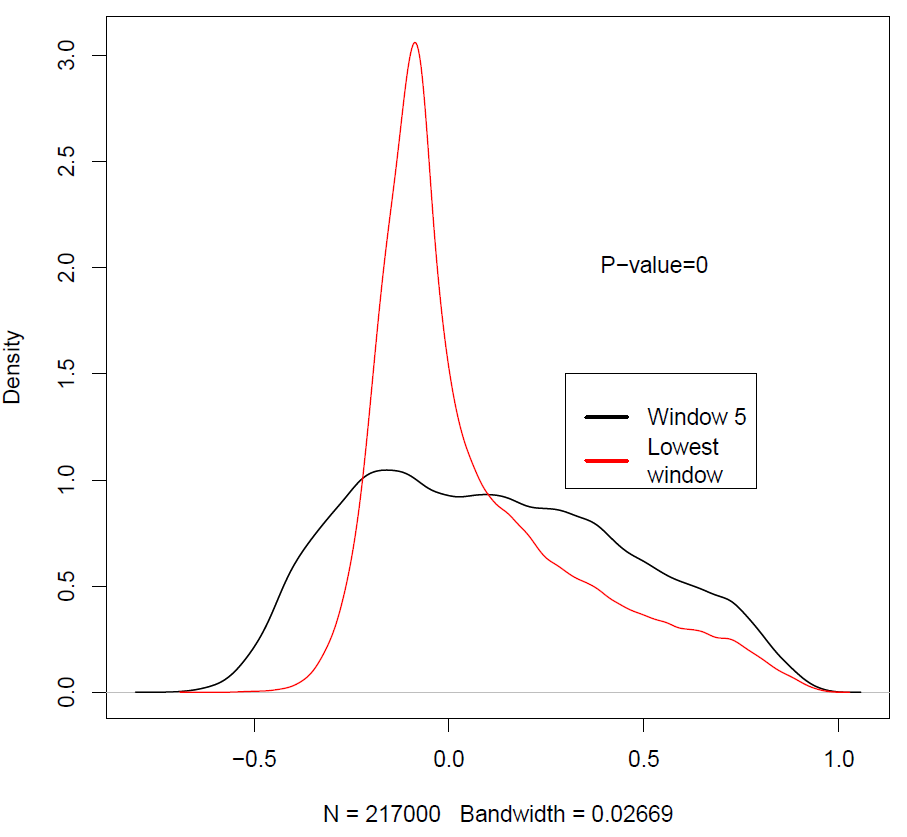 | 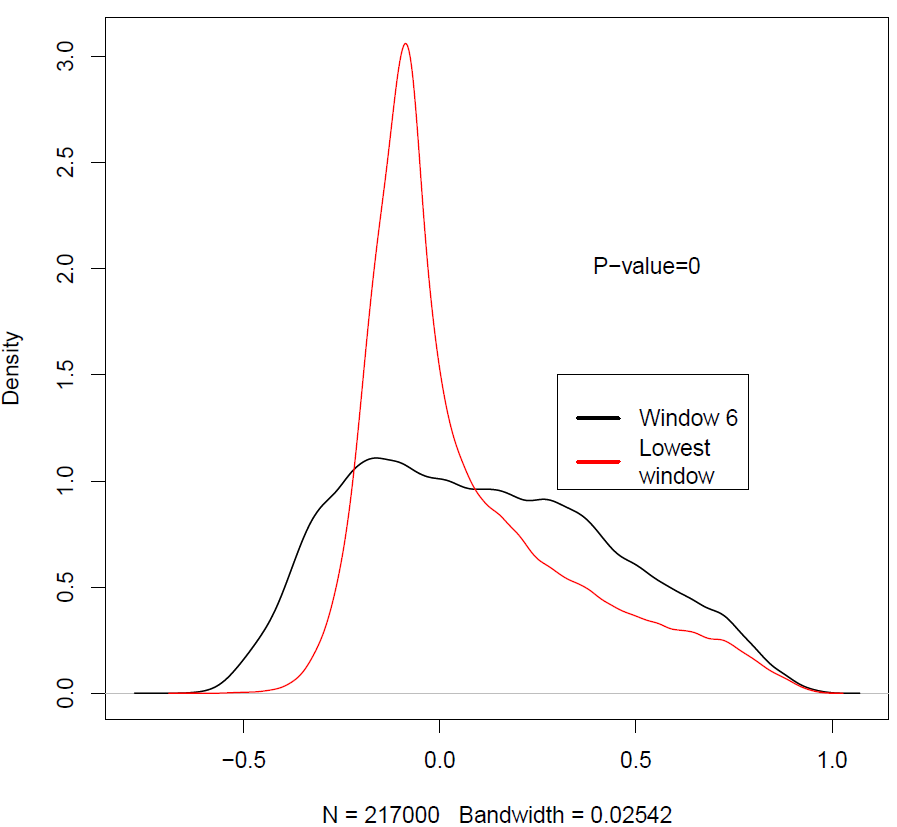 |
| 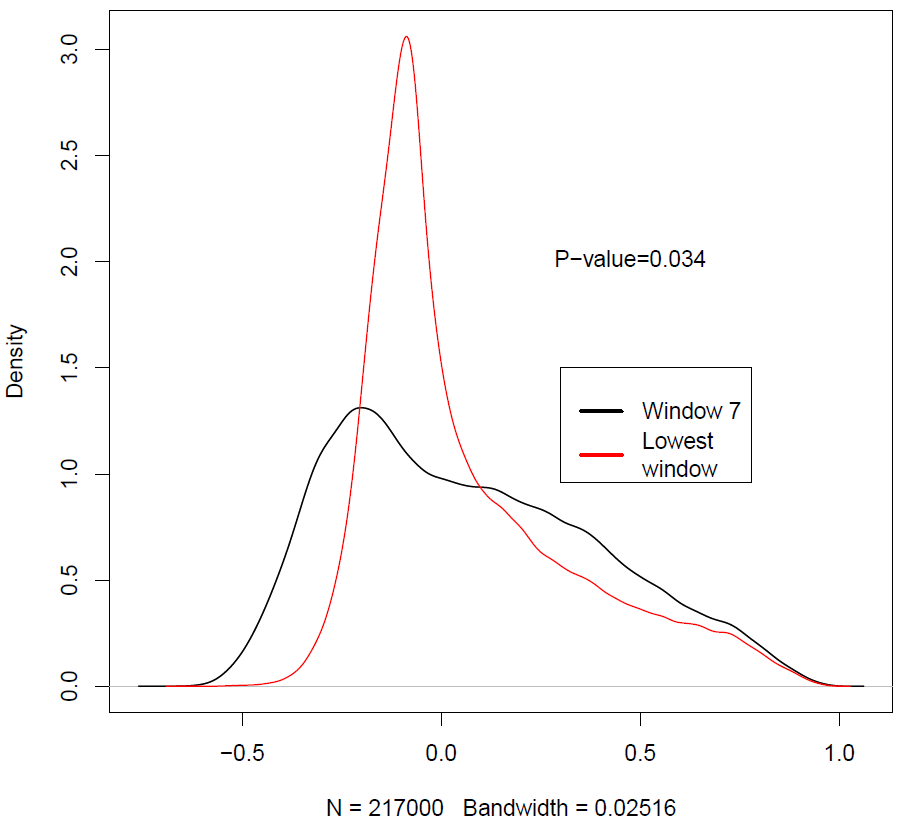 | 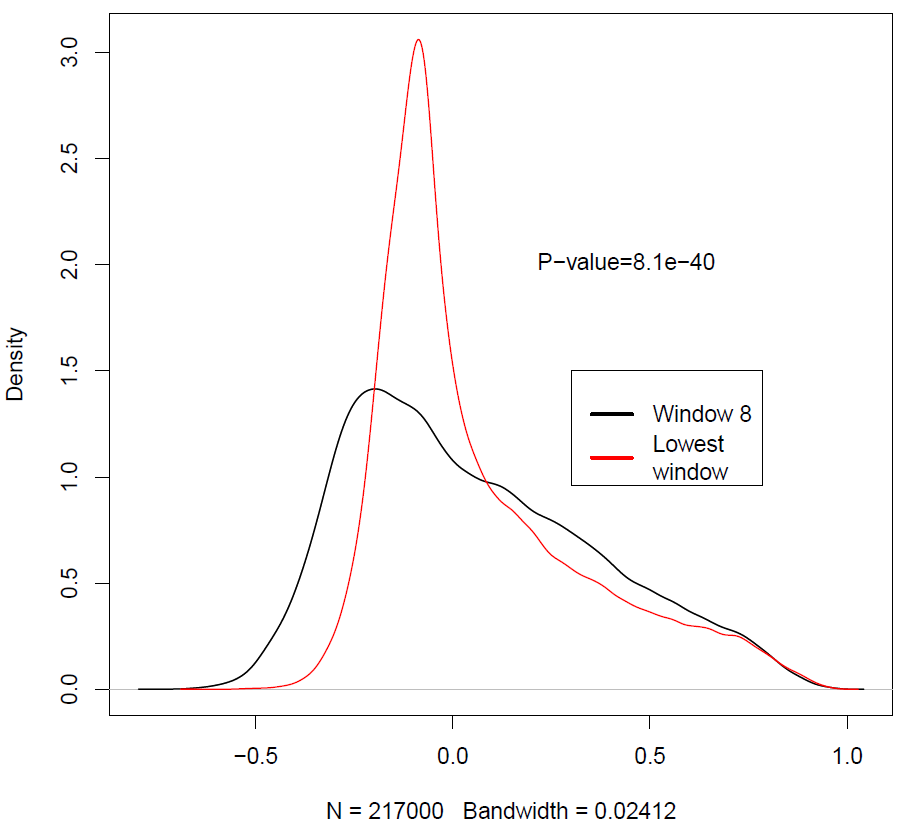 |
| 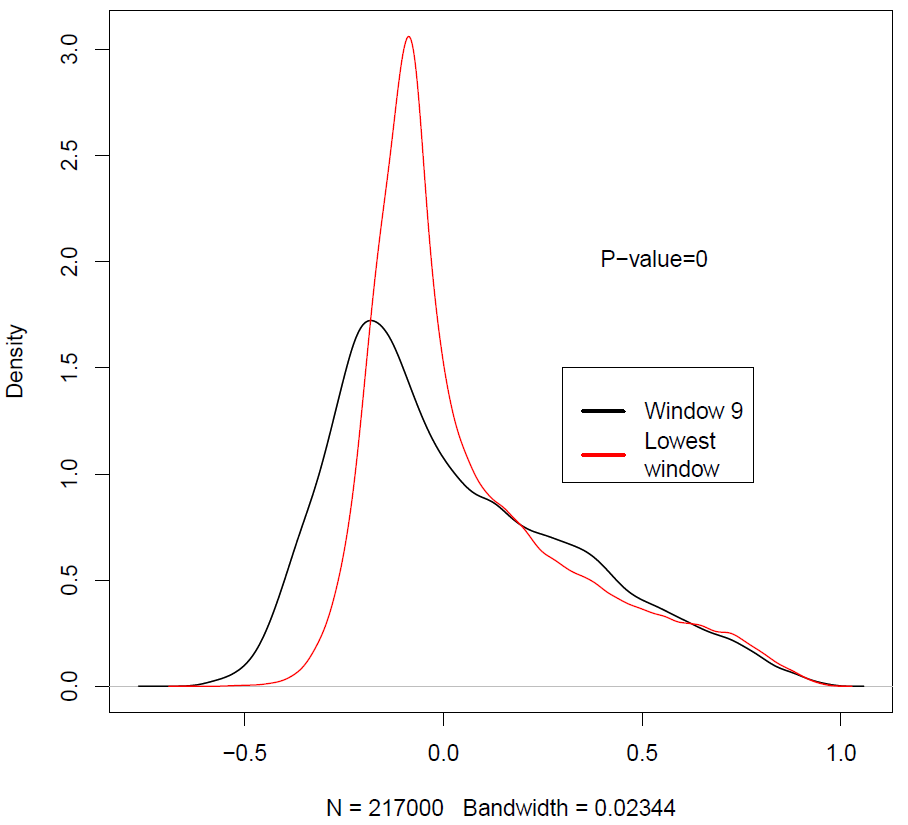 | 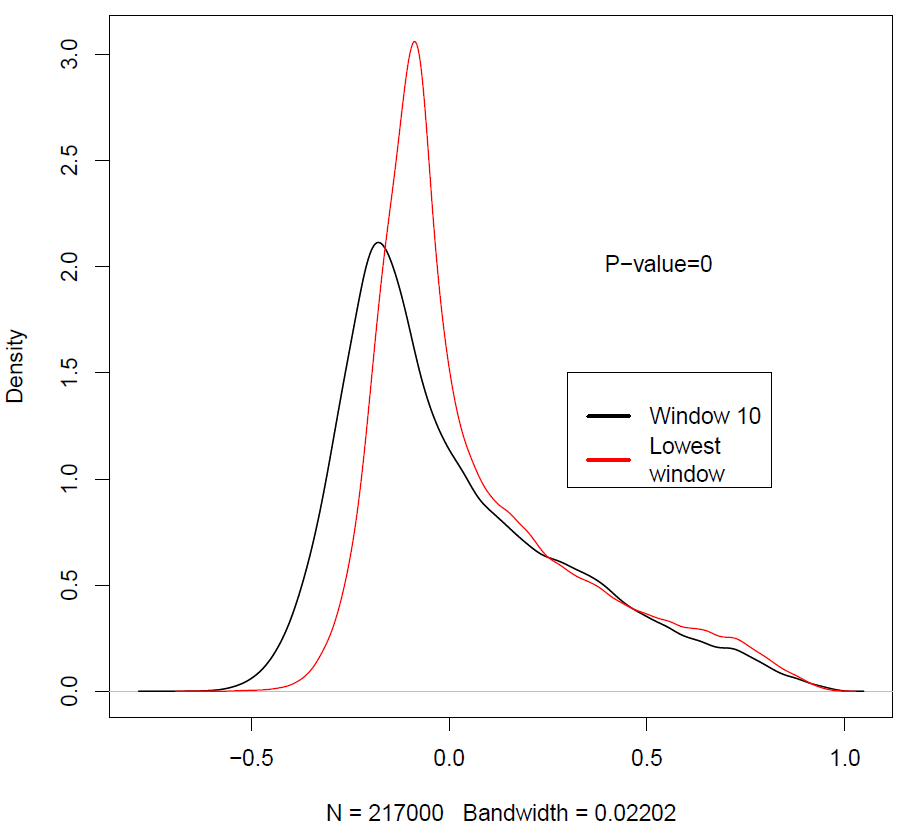 |
| 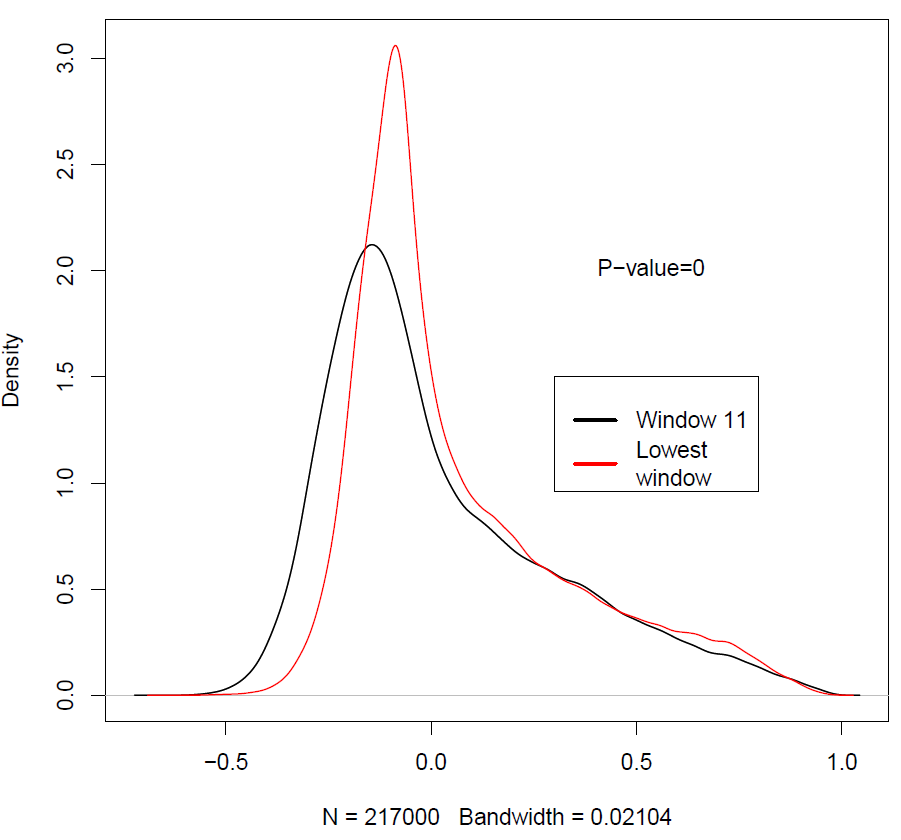 | 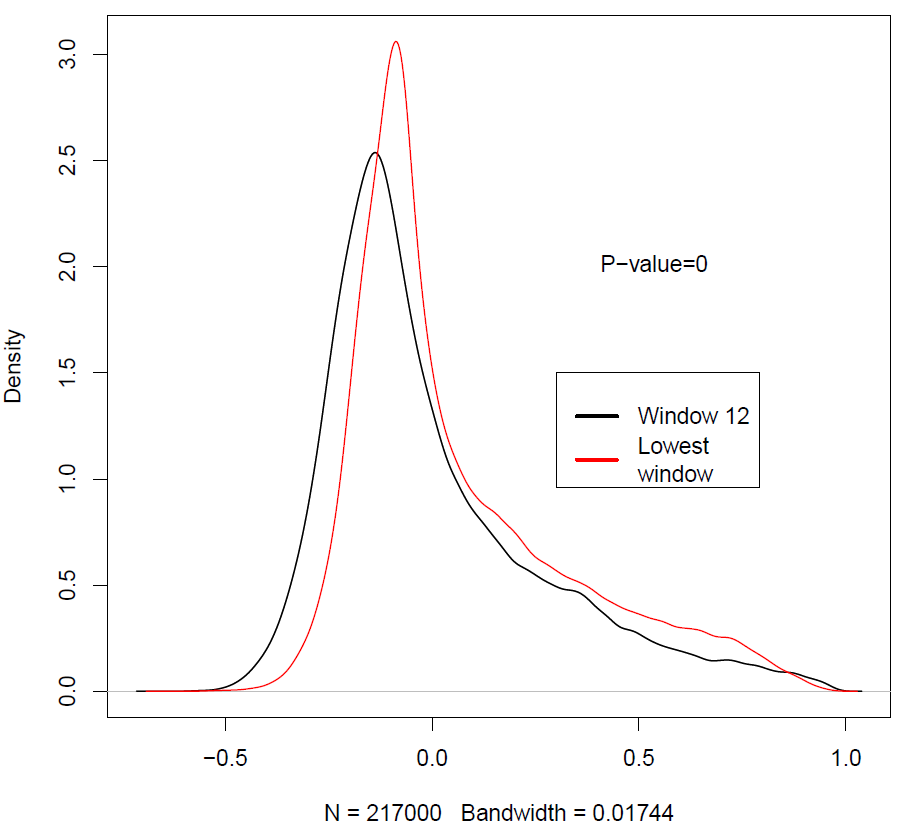 |
| 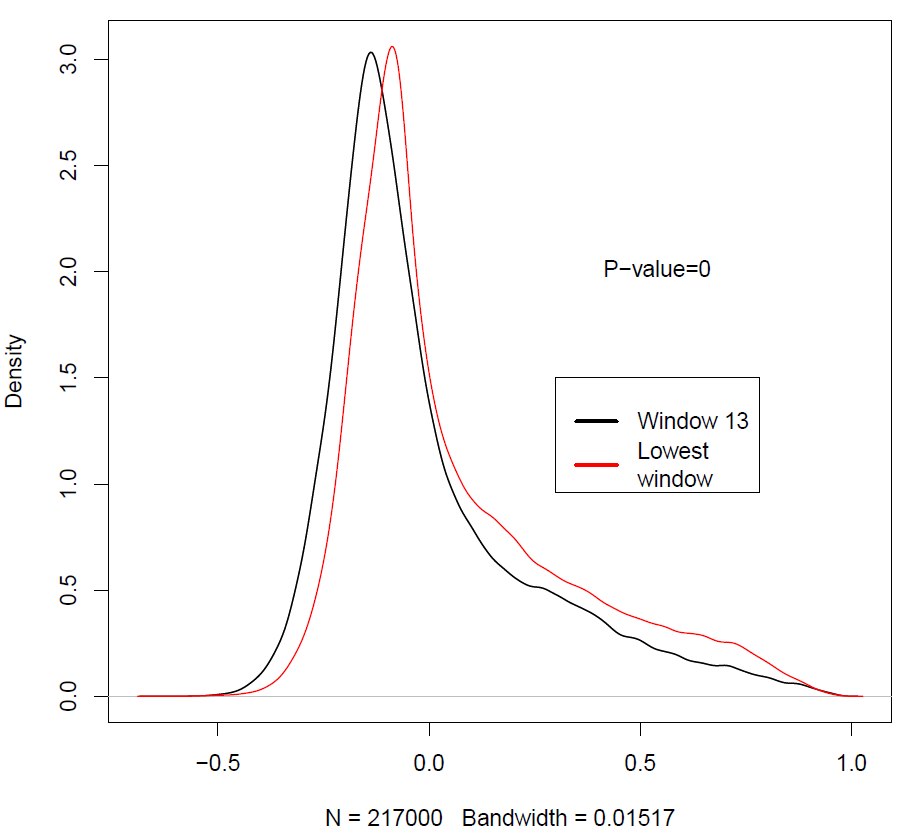 | 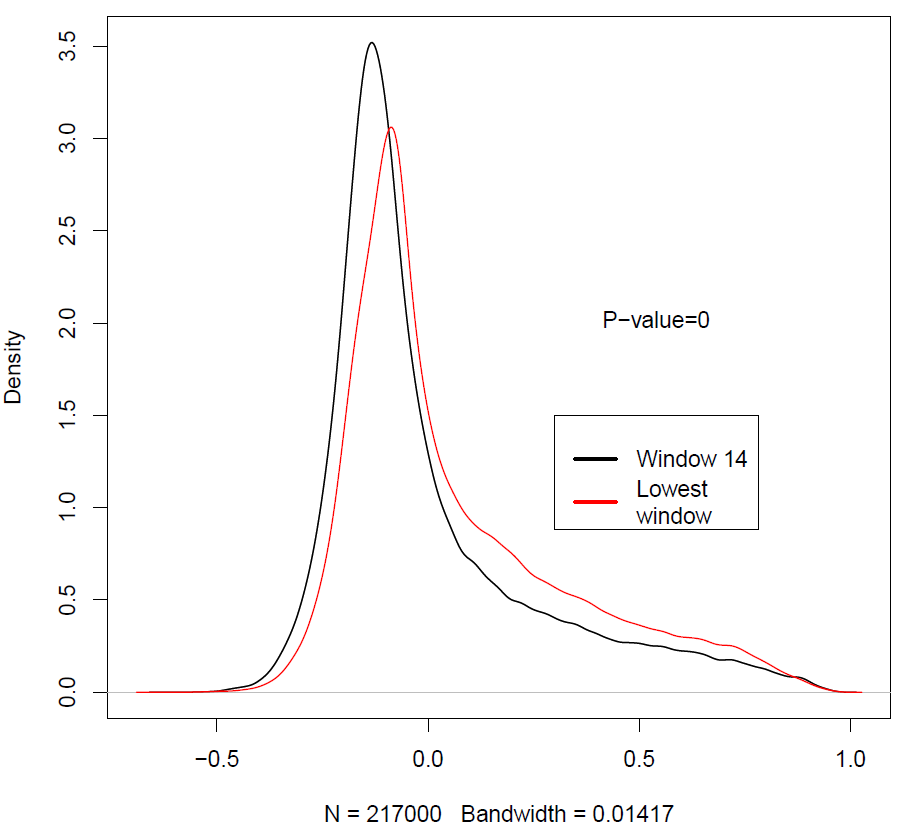 |
| 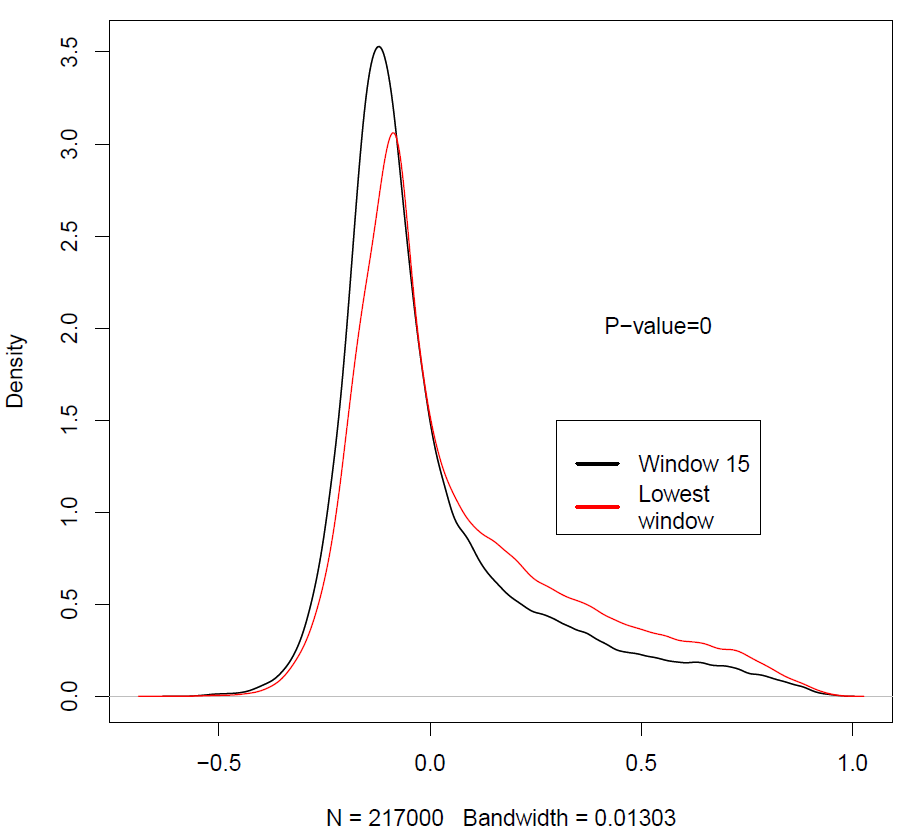 |  |

**Figure S2.** Distributions of correlation of high expression genes with genes in 18 windows of mean expression compared with the lowest mean expression window, in Human ES cells. The genes of Windows 1 to 4 were chosen as the ones that are above the threshold of technical variation.


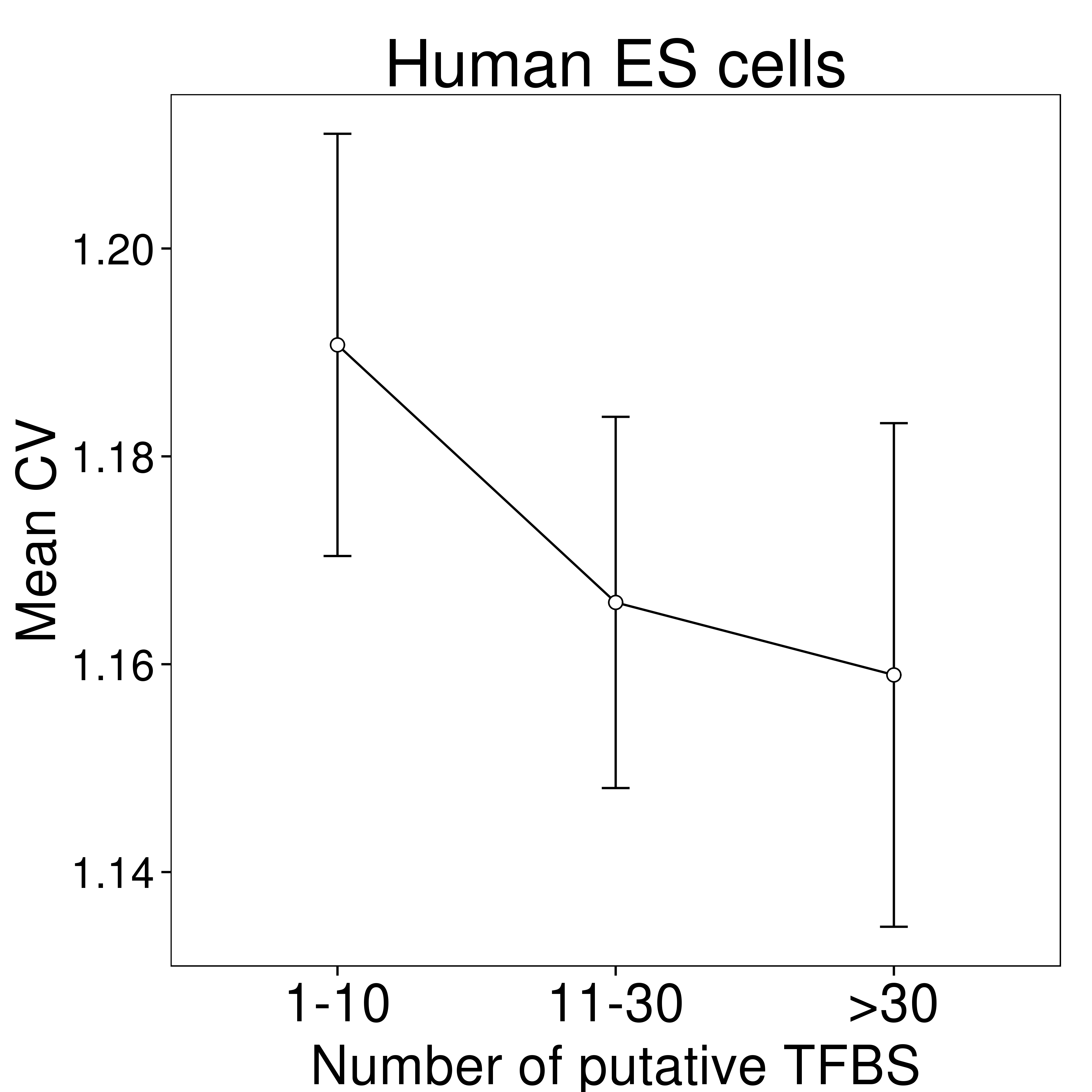


**Figure S3.** Number of putative Transcription Factor Binding Sites (TFBS) per gene (shown in 3 bins) and their corresponding Mean CV values. There was no statistically significant difference between means


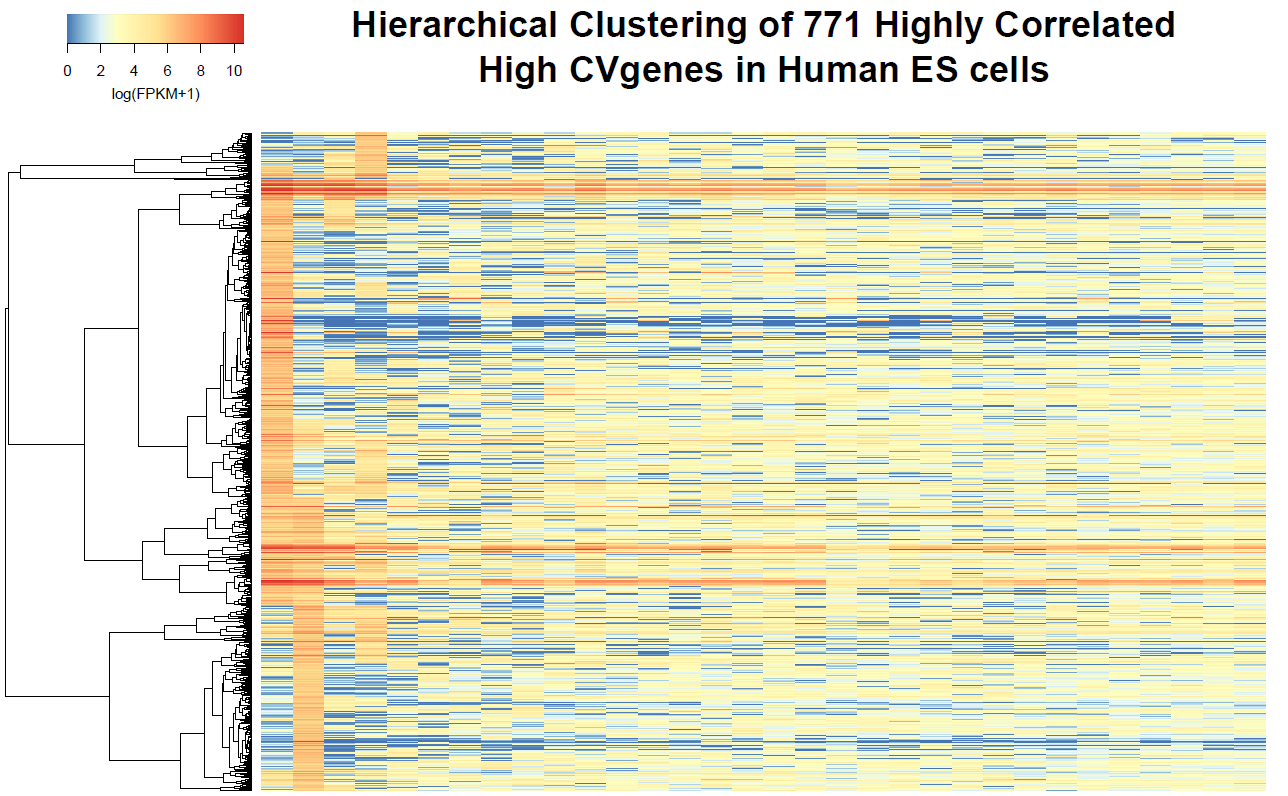


**Figure S4.** Heatmap of gene expression values (in log(FPKM+1)) of highly correlated variable (High CV) genes in Mouse ES cells.


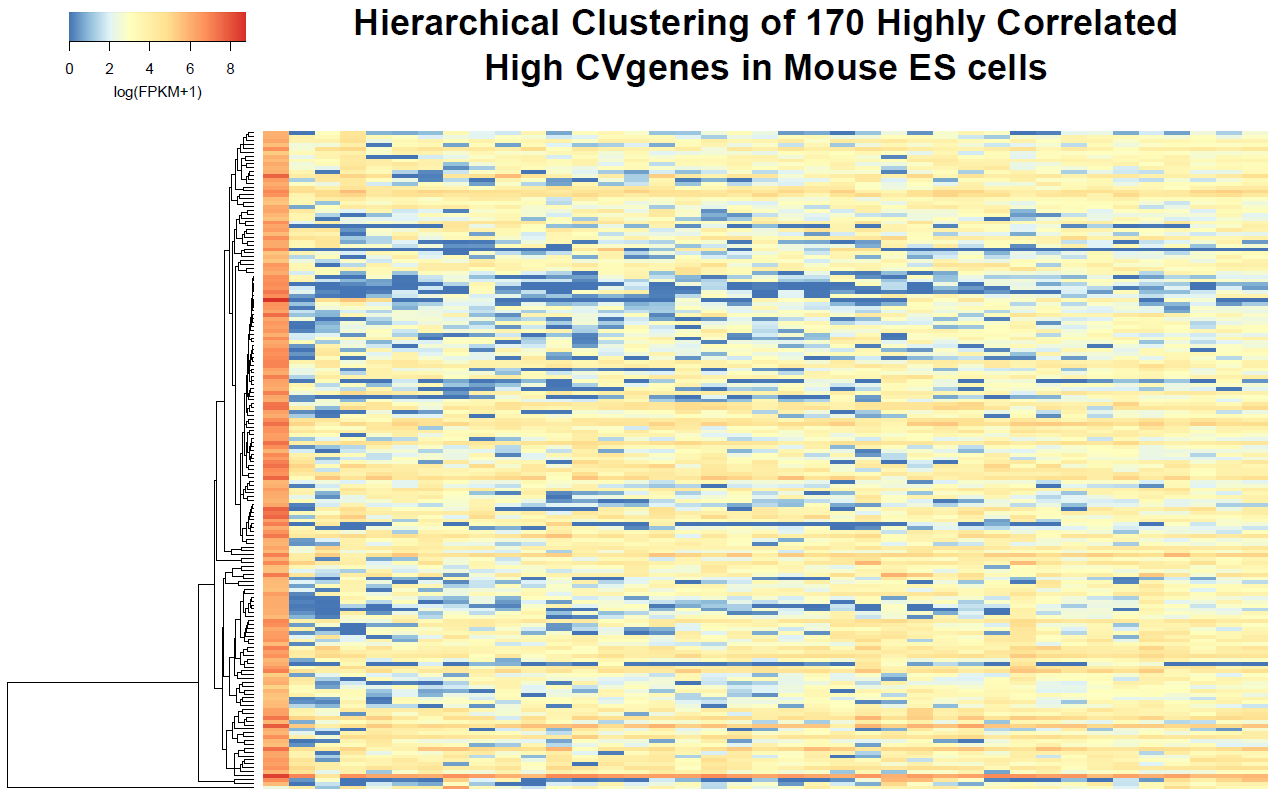


**Figure S5.** Heatmap of gene expression values (in log(FPKM+1)) of highly correlated variable (High CV) genes in Human ES cells.


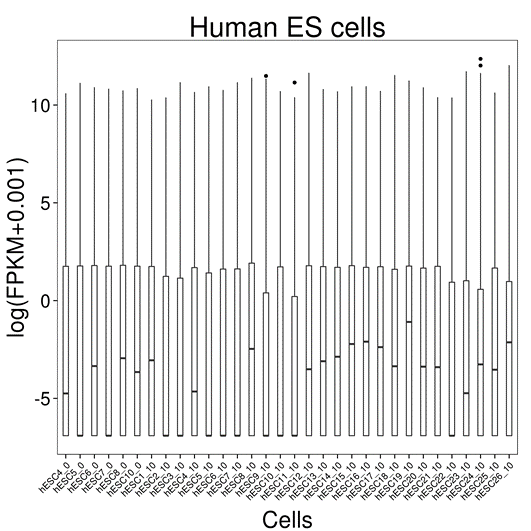


**Figure S6.** Boxplot of FPKM values for all cells in human


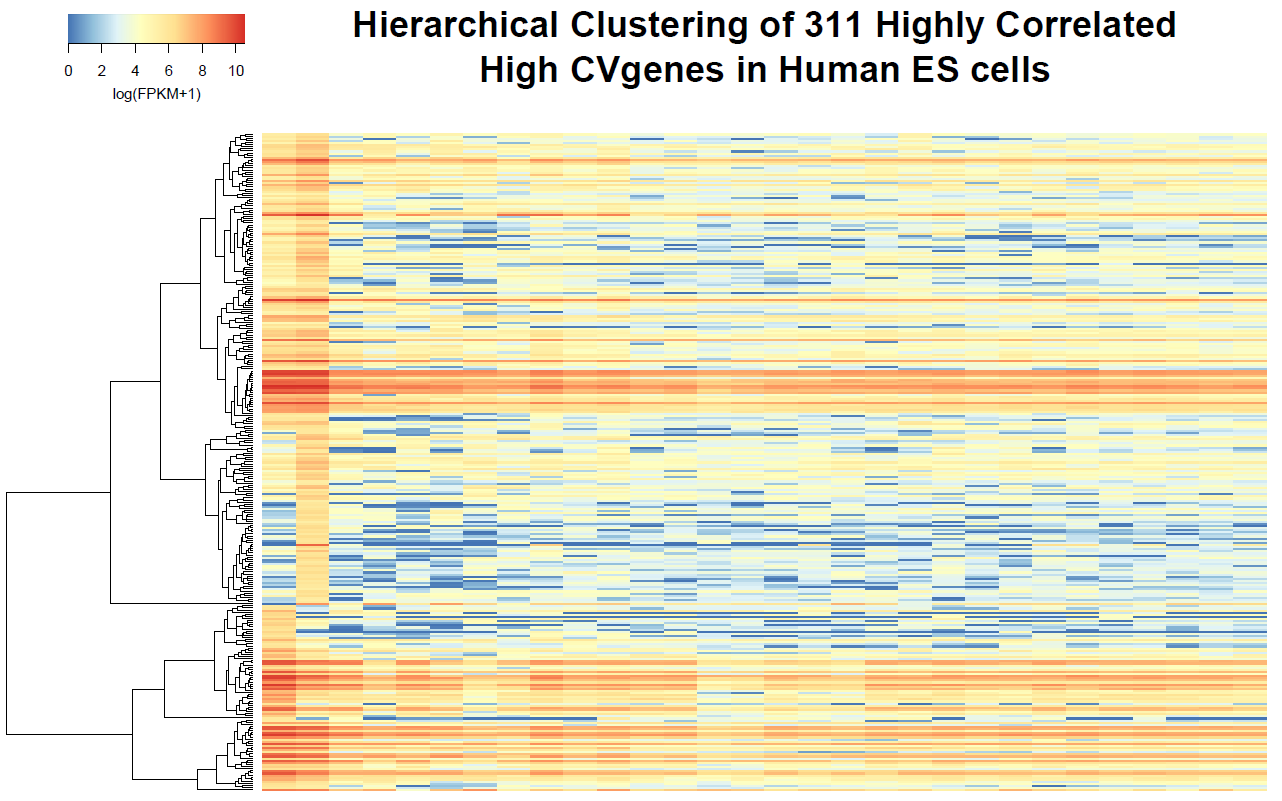


**Figure S7.** Heatmap of High CV genes in Human ES cells after discarding cells 24 and 26
